# *TIMP3* and *TIMP1* are risk genes for bicuspid aortic valve and aortopathy in Turner syndrome

**DOI:** 10.1371/journal.pgen.1007692

**Published:** 2018-10-03

**Authors:** Holly Corbitt, Shaine A. Morris, Claus H. Gravholt, Kristian H. Mortensen, Rebecca Tippner-Hedges, Michael Silberbach, Cheryl L. Maslen, GenTAC Registry Investigators

**Affiliations:** 1 Knight Cardiovascular Institute, Oregon Health & Science University, Portland, Oregon, United States of America; 2 Department of Molecular and Medical Genetics, Oregon Health & Science University, Portland, Oregon, United States of America; 3 Department of Pediatrics, Division of Pediatric Cardiology, Baylor College of Medicine, Houston, Texas, United States of America; 4 Department of Endocrinology and Internal Medicine and Medical Research Laboratories, Aarhus University Hospital, Aarhus, Denmark; 5 Department of Molecular Medicine, Aarhus University Hospital, Aarhus, Denmark; 6 Cardiorespiratory Unit, Great Ormond Street Hospital for Children, London, United Kingdom; 7 Department of Pediatrics, Division of Pediatric Cardiology, Oregon Health & Science University, Portland, Oregon, United States of America; Albert Einstein College of Medicine, UNITED STATES

## Abstract

Turner syndrome is caused by complete or partial loss of the second sex chromosome, occurring in ~1 in 2,000 female births. There is a greatly increased incidence of aortopathy of unknown etiology, including bicuspid aortic valve (BAV), thoracic aortic aneurysms, aortic dissection and rupture. We performed whole exome sequencing on 188 Turner syndrome participants from the National Registry of Genetically Triggered Thoracic Aortic Aneurysms and Cardiovascular Related Conditions (GenTAC). A gene-based burden test, the optimal sequence kernel association test (SKAT-O), was used to evaluate the data with BAV and aortic dimension z-scores as covariates. Genes on chromosome Xp were analyzed for the potential to contribute to aortopathy when hemizygous. Exome analysis revealed that *TIMP3* was associated with indices of aortopathy at exome-wide significance (p = 2.27 x 10^−7^), which was replicated in a separate cohort. The analysis of Xp genes revealed that *TIMP1*, which is a functionally redundant paralogue of *TIMP3*, was hemizygous in >50% of our discovery cohort and that having only one copy of *TIMP1* increased the odds of having aortopathy (OR = 9.76, 95% CI = 1.91–178.80, p = 0.029). The combinatorial effect of a single copy of *TIMP1* and *TIMP3* risk alleles further increased the risk for aortopathy (OR = 12.86, 95% CI = 2.57–99.39, p = 0.004). The products of genes encoding tissue inhibitors of matrix metalloproteinases (TIMPs) are involved in development of the aortic valve and protect tissue integrity of the aorta. We propose that the combination of X chromosome *TIMP1* hemizygosity and variants of its autosomal paralogue *TIMP3*, significantly increases the risk of aortopathy in Turner syndrome.

## Author summary

BAV is the most frequent congenital heart defect, occurring in about 1–2% of the population with 70% of cases occurring in males. BAV increases risk for thoracic aortic aneurysm (TAA) and early death. Approximately 30% of individuals with Turner syndrome have BAV/TAA, making this an important population for the study of this disease. Given that individuals with Turner syndrome are missing a complete or partial second sex chromosome, it is presumed that X chromosome genes are involved in causing the defect. This is consistent with the bias towards occurrence in euploid males. However, not everyone with Turner syndrome has a BAV, so we hypothesized that autosomal genes may also play a role. Using whole exome sequencing we have shown that deleterious variation in *TIMP3* is associated with BAV and indices of TAA. We further found that there is a synergistic interaction between loss of the X chromosome gene, *TIMP1*, and deleterious variation in *TIMP3* that significantly increases that risk. TIMP1 and TIMP3 play roles in aortic valve morphogenesis and in stabilizing the aortic wall, loss of which leads to TAA. Hence our findings have implications for understanding the cause of BAV/TAA in all populations and as a potential therapeutic target.

## Introduction

Turner syndrome is the most common sex chromosome aneuploidy, where ~50% have a complete monosomy X and ~48% have either a partial loss, rearrangement, or mosaicism of a second X chromosome.[1] The remaining ~2% have a partial or mosaic Y chromosome. Although Turner syndrome can be compatible with life, less than 1% of Turner syndrome fetuses survive.[2] The majority of prenatal deaths are due to cardiovascular defects.[3] Live born females with Turner syndrome share a constellation of phenotypes including primary ovarian insufficiency, short stature, lymphedema, webbed neck, skeletal deformities, neurocognitive disability, and a high incidence of congenital cardiovascular malformations. In particular, they are at a greatly increased risk for having left heart obstructions including hypoplastic left heart syndrome, BAV, coarctation of the aorta, and TAA.[4] Heart defects are the major cause of premature death. The degree to which a second sex chromosome is retained is the primary determinant of the morbidity and mortality in Turner syndrome, an observation that strongly implicates X chromosomal genetics in the pathology of acquired and congenital cardiovascular disease.[5, 6] In depth studies have shown that BAV, coarctation of the aorta, and risk for aneurysm are linked to the short arm of the X chromosome (Xp).[7, 8]

BAV is a congenital malformation where the aortic valve is comprised of two leaflets as opposed to the normal three leaflet configuration. BAV is associated with lifelong heart disease including valve calcification, stenosis, aortic endocarditis, and thoracic aortic dilation (TAD) that has a high risk of progression to aneurysm, dissection and rupture, and premature death. It is the most common congenital heart malformation occurring in about 2% of the general population where it is predominantly found in males, which comprise about 70% of all BAV cases.[9] However, despite the prevalence in the population, little is known about the etiology of BAV. There is clearly a genetic component as 10–40% of BAV is familial.[10] BAV and aortic aneurysm are thought to have a common genetic etiology.[11] Mutations in *NOTCH1*[12], *GATA5*[13], and *NKX2.5*[14] have been identified as the causative factor in some families with inherited BAV, but the majority of cases remain unexplained. The sex bias in euploid BAV indicates that having two X chromosomes may be protective. In Turner syndrome the incidence of BAV is increased by at least 50-fold over that seen in the euploid population.[15] This suggests that the lack of a second X chromosome predisposes both males and Turner syndrome females to have BAV and TAA, a condition known as BAV aortopathy.

Although there is a paucity of information about the etiology for BAV, a great deal is known about the pathogenic events underlying TAA and dissections associated with BAV. Numerous studies have shown significantly increased expression of matrix metalloproteinases (MMPs) and decreased expression of TIMPs in aneurysmal tissue.[16] This is significant because the role of MMPs is to degrade extracellular matrix (ECM); an activity that is inhibited by TIMPs. It is thought that in aneurysms the ECM in the aortic wall becomes degraded by MMPs, which weakens the aorta allowing it to succumb to hemodynamic stress thereby enlarging the diameter and thinning the aortic wall. In particular, increased expression of MMP2 and MMP9, which degrade the collagen and elastin components of the aortic wall, and a decrease in TIMP1, which inhibits MMP2 and MMP9 activity, have been implicated in the pathogenesis of aortic aneurysms.[16] In addition, an increased MMP9/TIMP1 ratio has been shown to be elevated in chronic aortic dissection, demonstrating a persistent role for ECM degradation.[17]

Deficiency of the second sex chromosome contributes to aortopathy in Turner syndrome, but its loss is not sufficient to cause disease since ~50% of women with Turner syndrome have a normal aortic valve and aortic dimensions. We hypothesized that autosomal genetic variation sensitized by sex chromosome deficiency causes aortopathy in Turner syndrome. To address this hypothesis we used whole exome sequencing to identify autosomal genetic variation associated with BAV and TAD in Turner syndrome. We used TAD as an indicator of aneurysm formation. This study of a discovery cohort of 188 and a replication cohort of 53 individuals with Turner syndrome identified an exome-wide significant association between *TIMP3* (MIM: 188826) and BAV/TAD. Furthermore, investigation of the *TIMP3* paralog, *TIMP1* (MIM: 305370), revealed that having more than one copy of the Xp chromosome gene *TIMP1* was protective against BAV/TAD. Combinatorial analysis shows a synergistic effect between having a single copy of *TIMP1* plus the *TIMP3* risk allele and the occurrence of BAV/TAD. Knowledge of a direct link between TIMP family-gene expression and aortopathy points the way to the development of novel biomarkers for disease progression and therapies to combat catastrophic aortic dissection and rupture in Turner syndrome.

## Results

### Correlation of aortic enlargement with BAV

**Fig 1:**
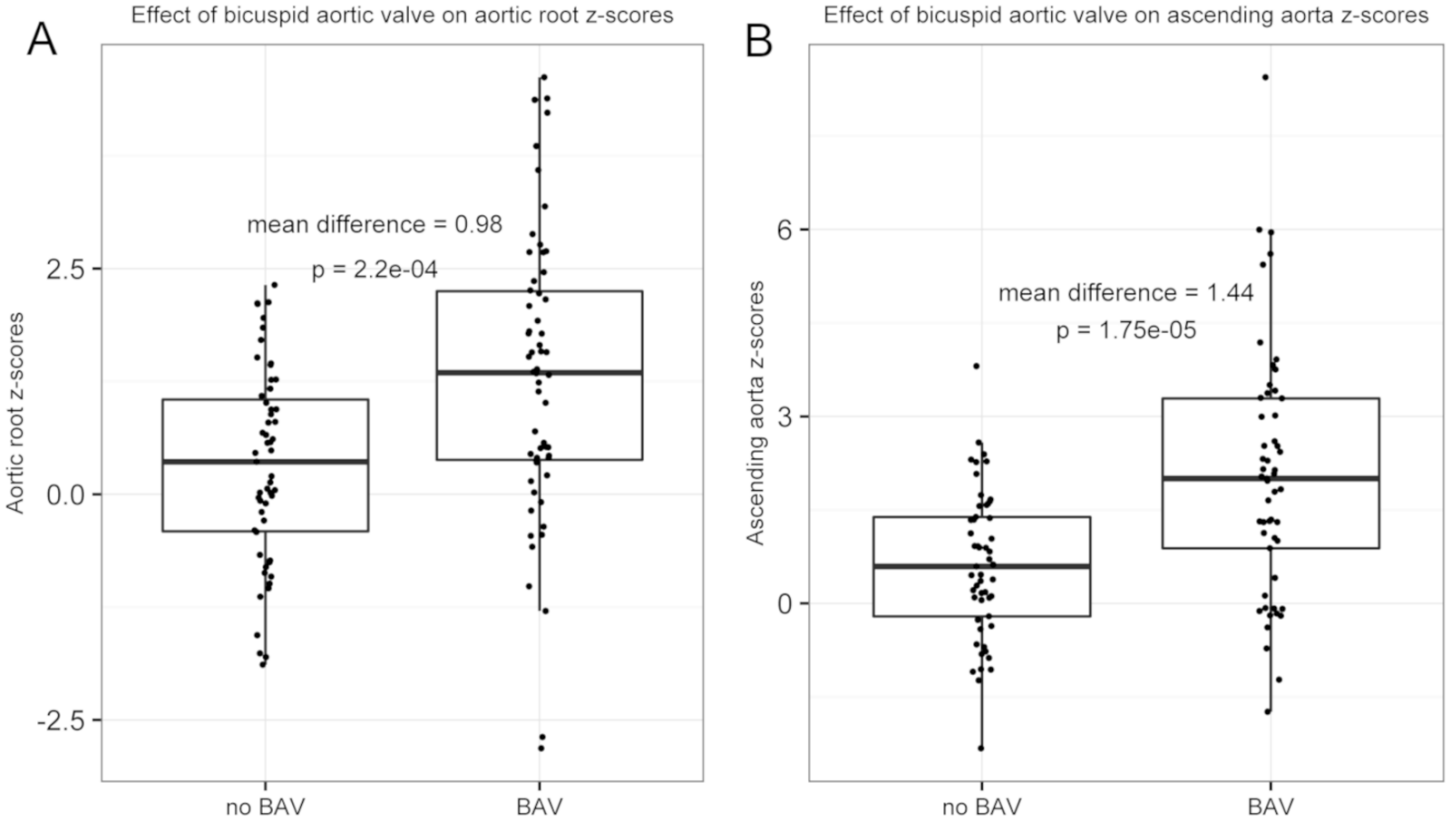
Aortic z-scores are associated with BAV in the Turner syndrome discovery cohort. Box plot of **A)** AR z-scores and **B)** AAO z-scores for individuals with and without a BAV, where both were significantly associated with the presence of a BAV, p = 0.0002 and p<0.0001, respectively.

The presence of BAV was associated with a higher aortic root (AR) z-score (mean AR z-score in BAV 1.29 ±1.59, versus no BAV 0.31 ± 1.08, p = 0.0002, mean difference = 0.98; Fig 1A). BAV was also associated with a significantly higher ascending aorta (AAO) z-score (mean AAO z-score in BAV 2.04 ± 1.99, versus no BAV 0.61 ± 1.18, p<0.0001, mean difference = 1.44; Fig 1B).

### Association of TIMP3 with BAV and aortic dilation

**Fig 2:**
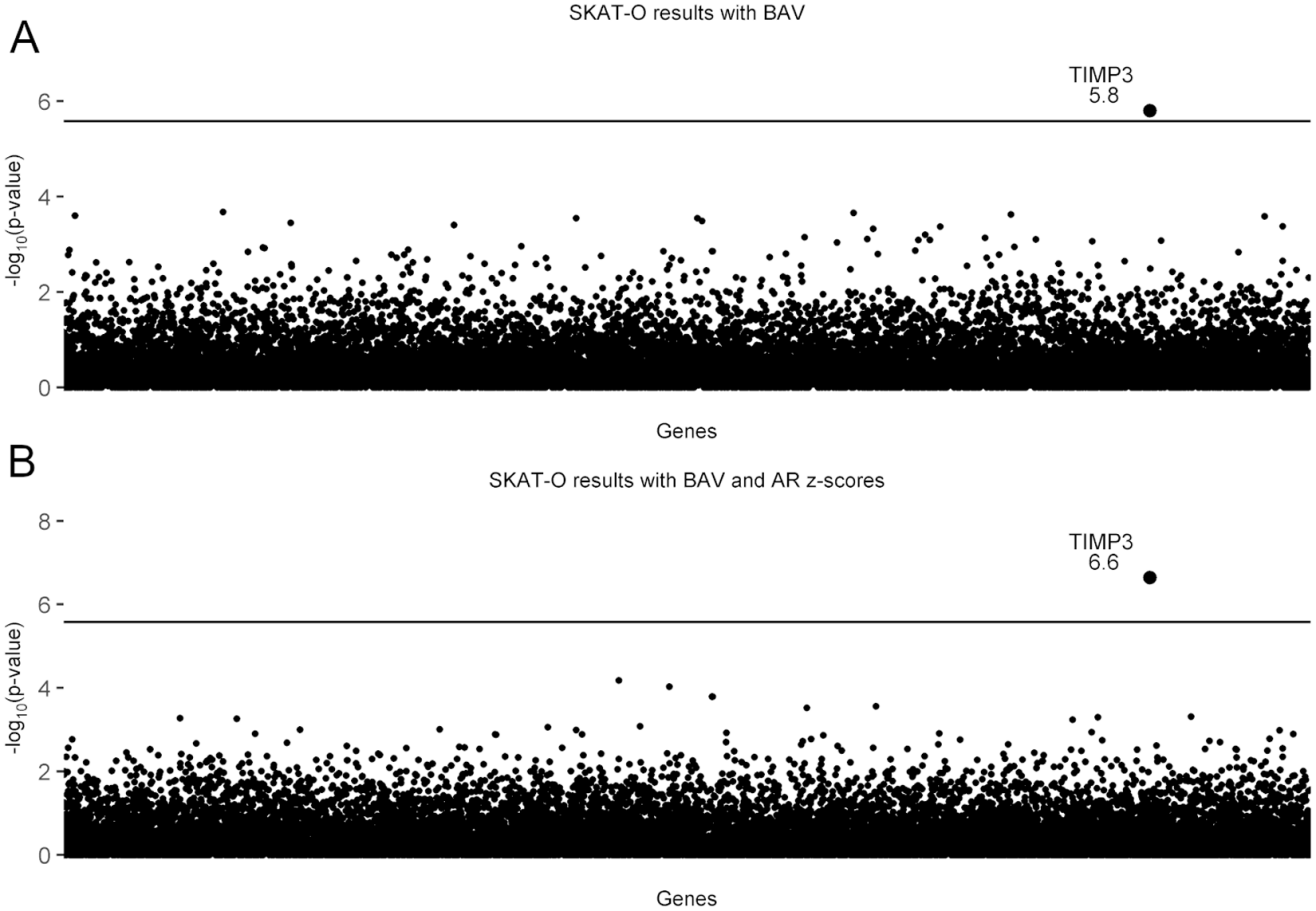
SKAT-O analysis shows that *TIMP3* variants are associated with BAV/TAD. Manhattan plots showing the exome-wide significant finding that *TIMP3* variants are associated with BAV, and with AR enlargement as an indicator of TAD. The horizontal line is the threshold for exome-wide significance (based on testing 19,392 genes, the exome-wide significance p-value = 2.578x10^-6^). *TIMP3* is the only gene that exceeds exome-wide significance. It is notable that no other genes approach the significance line. **A**) Shows the association with BAV as the sole predictor (p = 1.58x10^-6^). **B**) Shows the association results for BAV and aortic root (AR) z-scores as covariates (p = 2.27x10^-7^). The significance level for *TIMP3* increases nearly 10-fold when AR z-scores were added.

SKAT-O analysis revealed that variants in *TIMP3* on chromosome 22 achieved exome-wide significance for association with BAV and TAD. *TIMP3* was associated with the occurrence of BAV when it was used as the sole dichotomous phenotype (p = 1.58x10^-6^; Fig 2A), with the significance level increasing by an order of magnitude when BAV and AR z-scores were evaluated as covariates (p = 2.27x10^-7^; Fig 2B). This demonstrates a *TIMP3*-driven association between BAV and aortic enlargement in Turner syndrome. The quantile-quantile plots showed that there was no departure from observed vs. expected p-values (S1 Fig). Targeted exome sequencing of *TIMP3* in a replication cohort also showed a significant association of *TIMP3* variants with BAV and AR z-scores using SKAT-O (p = 0.038; Table 1).

**Table 1:** Replication cohort sequencing results.

**Chromosome**	**Position**	**ID**	**REF**	**ALT**	**MAF cases**	**MAF controls**
22	33253280	rs9862	T	C	50.00%	50.00%
22	33253292	rs11547635	C	T	14.29%	6.40%
22	33255244	rs149161075	C	T	3.57%	0.00%
**Gene**	**P value**	**N variants in Test**				
*TIMP3*	0.03734*	3				

Variants identified in Danish cohort by Sanger sequencing of *TIMP3* exons.

ID; rs identifier from dbSNP

MAF; minor allele frequency

P value; calculated by SKAT-O gene-based association test

*; reaches the significance threshold of <0.05

N; number

### *TIMP3* rs11547635 is the major autosomal risk allele

There were a total of four variants identified in *TIMP3* in the discovery cohort (Table 2). Of the four variants, rs11547635 was determined to be the SNP predominantly driving the association based on the increased allele frequency in cases compared to controls (p = 0.001, chi-squared) and evidence that the variant is deleterious based on the CADD score of 16.67. This is above the recommended deleterious significance cutoff of 15, which indicates that is in the top 5% of all damaging variants in the human genome. On the gene level, *TIMP3* has a GDI PHRED score of 0.449, placing in the top 10% of genes intolerant of mutations. The lead driving SNP encodes a synonymous C>T transition at p.Ser87 in exon 3. Another SNP, rs9862, which is a synonymous variant at p.His83 is always present along with the p.Ser87 variant in the BAV cases in this study. Importantly, these variants, which have been studied in various types of cancer are associated with reduced TIMP3 plasma levels.[18–20] In combination the two variants disrupt two core ETS1 binding consensus sequences and prevent ETS1 binding, which is thought to be the basis of the reduction in expression.[20] Our discovery that known deleterious variants in *TIMP3* are significantly associated with BAV and TAD of the aortic root in Turner syndrome fits well with the known role for TIMPs in protection against aortopathy. Nearly 25% of our Turner syndrome cohort carry these SNPs, making them a significant risk genotype. The two additional *TIMP3* variants, rs149161075 and rs369072080, are rare and occur only in cases in this study.

**Table 2:** Summary of the *TIMP3* variants associated with BAV/TAD.

rsID	Protein Change	CADD Score	Expected Allele Frequency (Alleles/Total)	Observed Frequency in Cases (Cases/Total)	Observed Allele Frequency in Cases (Alleles/Total)	Observed Frequency in Controls (Controls/Total)	Observed Allele Frequency in Controls (Alleles/Total)
rs9862	p.His83 =	2.597	49.1% (32790/66734)	64.8% (57/88)	43.8% (77/176)	71.0% (71/100)	50.5% (101/200)
rs11547635	p.Ser87 =	16.67	7.1% (4735/66738)	23.8% (21/88)	11.9% (21/176)	6.0% (6/100)	3.5% (6/200)
rs149161075	p.Phe172 =	5.801	0.3% (203/66736)	2.3% (2/88)	1.1% (2/176)	0% (0/100)	0% (0/200)
rs369072080	p.Gly173 =	0.002	0% (0/66738)	1.1% (1/88)	0.6% (1/176)	0% (0/100)	0% (0/200)

All of the *TIMP3* variants identified through whole exome sequencing of subjects in our Turner discovery syndrome cohort are listed. The dbSNP rs identifier is listed, along with the consequence of the change, ExAC expected allele frequencies (European non-Finnish), CADD score, the number of subjects that had a BAV (case) or a normal valve (control), and the allele frequency of each variant, reported as percentages.

### *TIMP1* copy number influences aortopathy risk

**Fig 3:**
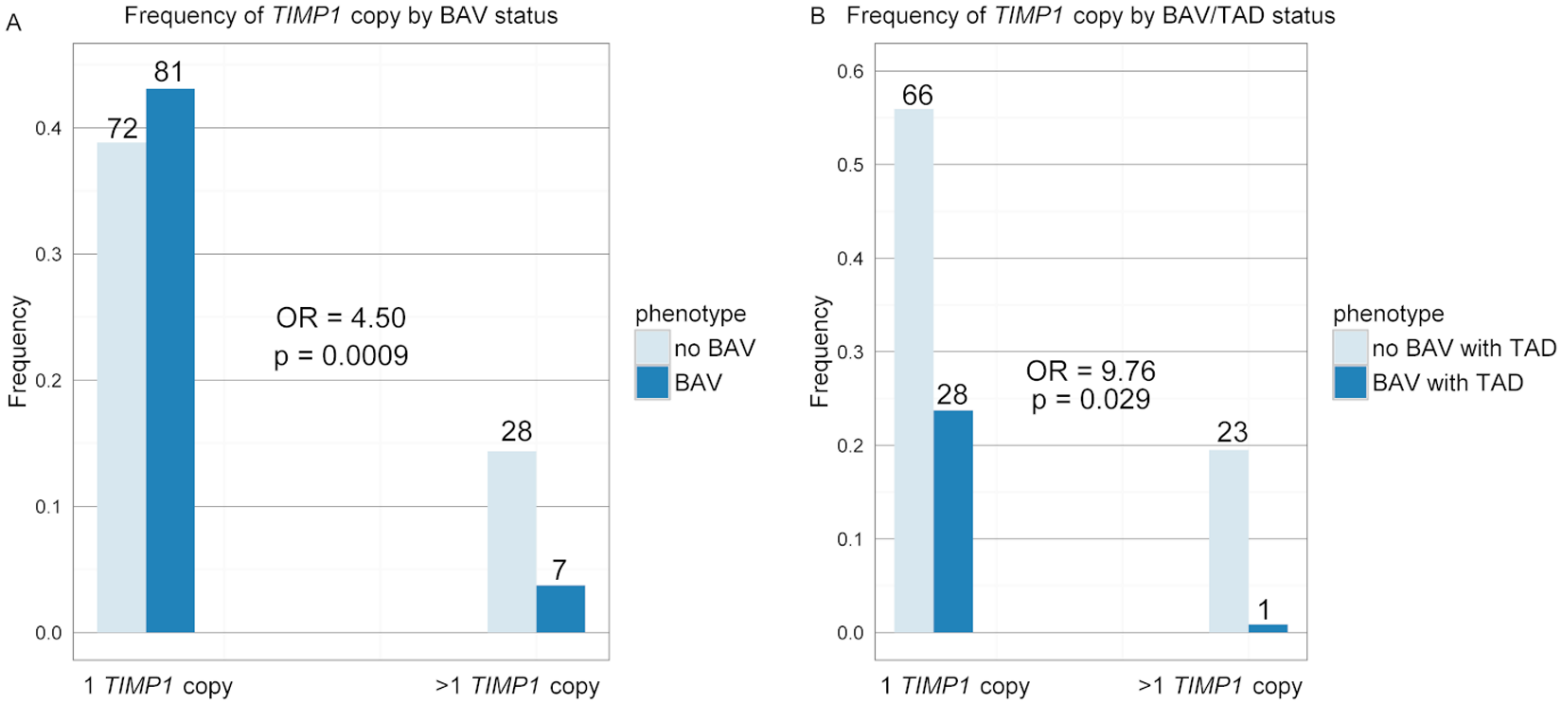
*TIMP1* copy number is associated with the risk of BAV and BAV with TAD. Bar graph showing the frequency of BAV with or without TAD when only one copy of *TIMP1* is present compared to the frequency when there is more than one copy of *TIMP1*. **A**) The dark blue bars represent individuals with a diagnosis of BAV and the light blue bars represent individuals without a BAV. **B**) The dark blue bars represent individuals with a diagnosis of BAV with TAD and the light blue bars represent individuals without a BAV with TAD. For both a logistic regression model was run, with *TIMP1* copy number as the categorical predictor and the phenotype as the response variable. Odds ratio and p-value were calculated and showed that having a single copy of *TIMP1* significantly increased the odds of having a BAV and a BAV with TAD.

Analysis of all of the genes on Xp identified *TIMP1* as the top gene meeting our aortopathy criteria, which includes the potential for escape from X-inactivation, no Y chromosome or autosome homologues, and expression in the aorta. The list of all of the genes that met the criteria is shown in Table 3, ranked according to the likelihood that they could contribute to aortopathy. The list of all Xp genes and their characteristics can be found in S1 Table. *TIMP1* polymorphically escapes X inactivation,[21] has partial functional redundancy with *TIMP3*[22], and is highly expressed in the aorta with nearly 10-fold higher expression than any of the other genes (GTExPortal). In addition, it is the only Xp gene that meets these criteria and has a known role in aortic valve development.[23] *TIMP1* is also the only gene on Xp with a known association with aortic aneurysms in both humans and mouse models. *Timp1* mouse models are susceptible to the development of aortic aneurysms[24, 25] and TIMP1 is known to be reduced in TAA in humans.[16, 26] Additionally, overexpression of Timp1 prevents aneurysm degradation and rupture in a rat model.[27] We therefore hypothesized that reduced copy number of *TIMP1* in Turner syndrome increases the risk for BAV/TAD. Using BAV as the only variable the analysis revealed that subjects with only one copy of *TIMP1* have a 4.50 increased odds of having a BAV than those who have greater than one copy (p = 0.0009, 95% CI = 1.9–11.8, Fig 3A). When BAV with TAD was studied as the outcome, having only one copy of *TIMP1* increased these odds substantially (OR = 9.76, p = 0.029, CI = 1.91–178.80, Fig 3B).

**Table 3:** Xp genes that meet criteria for being involved in BAV/TAD.

Gene	ensembl	cdsStart—cdsEnd*	X inactivation status	Pseudogene/ Y homolog	Aorta Expression (GTEx median RPKM)	Valve Development	Rank
***TIMP1***	**ENSG00000102265**	**X:47442814–47446090**	**Variable**	**No**	**423.6**	**Yes**^**26**^	**1**
*RBM3*	ENSG00000102317	X:48433568–48435474	Variable	No	48.96	unknown	2
*UBA1*	ENSG00000130985	X:47058201–47074328	Active	No	41.44	unknown	3
*INE2*	ENSG00000281371	X:15805712–15805712	Active	No	12.36	unknown	4
*AP1S2*	ENSG00000182287	X:15845447–15870647	Mostly Active	No	8.701	unknown	5
*GEMIN8*	ENSG00000046647	X:14027031–14039597	Mostly Active	No	6.69	unknown	6
*CA5B*	ENSG00000169239	X:15768146–15800787	Variable	No	5.198	unknown	7
*NHS*	ENSG00000188158	X:17653686–17750584	Mostly Active	No	4.852	unknown	8
*TRAPPC2*	ENSG00000196459	X:13732525–13738082	Mostly Active	No	4.289	unknown	9
*CA5BP1*	ENSG00000186312	X:15721474–15721474	Active	No	3.813	unknown	10
*CTPS2*	ENSG00000047230	X:16608915–16721025	Mostly Active	No	3.593	unknown	11
*INE1*	ENSG00000224975	X:47065254–47065254	Active	No	2.09	unknown	12
*TCEANC*	ENSG00000176896	X:13680627–13681683	Mostly Active	No	0.4852	unknown	13
*PPEF1*	ENSG00000086717	X:18725899–18845605	Variable	No	0.05353	unknown	14
*GRPR*	ENSG00000126010	X:16142076–16170768	Mostly Active	No	0.02957	unknown	15
*FAM9C*	ENSG00000187268	X:13056559–13061908	Active	No	0.005627	unknown	16

*Position on the X chromosome; cdsStart, start of coding sequence; cdsEnd, end of coding sequence, hg19

### *TIMP3* and *TIMP1*-associated risk is specific to the aorta

To determine the specificity of the association between *TIMP3* rs11547635 and *TIMP1* copy number for having BAV/TAD or other phenotypic features, we compared cases with or without rs11547635. Height, weight, blood pressure, body surface area, the presence of webbed neck, broad chest, primary ovarian insufficiency, hypertension, or lymphedema occurred with equal frequency in subjects with or without the rs11547635 SNP (Table 4). On the other hand BAV, BAV with TAD, coarctation of the aorta, and any aortic disease occurred with significantly higher frequency in the group with rs11547635, indicating that it is specifically associated with aortopathy. *TIMP1* copy number associations were similar but also included systolic blood pressure, lymphedema and webbed neck (Table 5).

**Table 4:** Attributes of Turner syndrome subjects with or without *TIMP3* rs11547635.

**Phenotype—Continuous**	**N**	**Mean with rs11547635**	**SD**	**Mean without rs11547635**	**SD**	**P value**		
Height (cm)	175	140.65	23.60	142.67	15.66	0.58		
Weight (kg)	168	56.07	21.10	55.15	20.30	0.84		
BSA (m^2^)	165	1.42	0.39	1.42	0.32	1		
BP, systolic	161	116.35	15.23	116.71	17.13	0.93		
BP, diastolic	161	68.39	11.97	72.20	10.59	0.12		
**Phenotype—Categorical**	**N**	**Affected with rs11547635**	**Unaffected with rs11547635**	**Affected without rs11547635**	**Unaffected without rs11547635**	**Odds Ratio**	**95% CI**	**P value**
Lymphedema	188	4	23	40	121	0.53	0.2–1.6	0.37
Broad chest	188	7	20	51	110	0.76	0.3–1.9	0.71
Webbed neck	188	16	11	69	92	1.94	0.8–4.4	0.17
POI^t^	154	12	79	10	53	0.81	0.3–2.0	0.82
Hypertension	178	11	16	60	91	1.04	0.5–2.4	0.92
Any Dissection	188	1	26	5	156	1.20	0.1–10.7	1.000^F^
Coarctation	188	12	15	33	128	3.10	1.3–7.3	0.014*
BAV	188	21	6	66	95	5.04	1.9–13.2	0.0008**
TAD^#^	118	9	9	29	71	2.45	0.9–6.8	0.140
BAV with TAD^#^	118	9	9	20	80	4.00	1.4–11.4	0.010^F^
BAV without TAD^#^	118	7	9	31	87	0.46	0.2–1.3	0.23^F^
Any aortic risk factor^~^	188	22	5	79	82	4.57	1.6–12.7	0.004**

Categorical p-value: chi-squared with yates correction.

^F^ Fishers exact test, Chi-square is calculated only if all expected cell frequencies are greater than or equal to 5

Continuous p-value: t-Test: Two-Sample Assuming Equal Variances

N, total number of subjects with data available for the category

^t^ POI, primary ovarian insufficiency. Excluded individuals under the age of 13 years.

^#^ Aortic root or ascending aorta (z-score > 1.9)

^~^ Aortic risk factors include BAV, coarctation, dilated aortic root or ascending aorta (z-score > 1.9)

^$^ For this group, the sample size was small and there were no affected individuals, so an accurate odds ratio could not be calculated.

*Significance level <0.05

**Significance level <0.005

**Table 5:** Attributes of Turner syndrome subjects with 1 or >1 copy of *TIMP1*.

**Phenotype—Continuous**	**N**	**Mean with 1 *TIMP1* copy**	**SD**	**Mean with >1 *TIMP1* copy**	**SD**		**P value**	
Height (cm)	175	142.58	15.55	141.53	22.85		0.75	
Weight (Kg)	168	55.79	20.12	53.22	22.13		0.52	
BSA (m^2^)	165	1.43	0.32	1.40	0.40		0.58	
BP, systolic	161	118.19	16.66	109.69	16.65		0.014*	
BP, diastolic	161	72.30	10.25	68.76	13.33		0.11	
**Phenotype—Categorical**	**N**	**Affected with 1 *TIMP1* copy**	**Controls with 1 *TIMP1* copy**	**Affected with >1 *TIMP1* copy**	**Controls with >1 *TIMP1* copy**	**Odds Ratio**	**95% CI**	**P value**
Lymphedema	188	43	110	1	34	13.29	1.8–100.2	0.003**
Broad chest	188	48	105	10	25	1.14	0.5–2.6	0.92
Webbed neck	188	77	76	8	27	3.42	1.5–8.0	0.006*
POI^t^	154	78	49	13	14	1.71	0.7–4.0	0.29
Hypertension	178	61	84	10	23	1.76	0.8–4.0	0.23
Any Dissection^$^	188	6	147	0	35	-	-	-
Coarctation	188	44	109	1	34	13.72	1.8–103.4	0.003**
BAV	188	81	72	7	28	4.50	1.9–11.8	0.0009**
TAD^#^	118	34	60	4	20	2.83	0.9–9.0	0.11
BAV with TAD^#^	118	28	66	1	23	9.76	1.3–75.8	0.019*
BAV without TAD^#^	118	27	67	4	20	2.01	0.6–6.5	0.34
Any aortic disease^~^	188	90	63	11	24	3.12	1.4–6.8	0.006**

Categorical p-value: chi-squared with yates correction.

^F^ Fishers exact test, Chi-square is calculated only if all expected cell frequencies are greater than or equal to

5Continuous p-value: t-Test: Two-Sample Assuming Equal Variances

N, total number of subjects with data available for the category

^t^ POI, primary ovarian insufficiency. Excluded individuals under the age of 13 years.

^#^ Aortic root or ascending aorta (z-score > 1.9)

^~^ Aortic risk factors include BAV, coarctation, dilated aortic root or ascending aorta (z-score > 1.9)

^$^ For this group, the sample size was small and there were no affected individuals, so an accurate odds ratio could not be calculated.

*Significance level <0.05

**Significance level <0.005

### Synergistic effect of *TIMP1* and *TIMP3* variation in aortopathy

We investigated the combinatorial effect of *TIMP1* and *TIMP3* variation on the outcome of BAV alone, and BAV with TAD. This analysis shows that the combination of having only one copy of *TIMP1* and being a carrier of *TIMP3* rs11547635 specifically increases the odds for having a BAV by nearly twenty-fold (OR = 18.00, 95% CI = 5.19–74.89, p<0.001) and also for having a BAV with TAD (OR = 12.86, 95% CI = 2.57–99.39, p = 0.004) compared to the group with no rs11547635 and >1 *TIMP1* (Table 6).

**Table 6:** Combinatorial effects of *TIMP1* copy number and *TIMP3* SNP rs11547635 on the outcome of BAV or BAV with TAD.

**Groups, outcome of BAV**	**N**	**N affected**	**% affected**	**OR**	**95% CI**	**P value**
no rs11547635 & >1 *TIMP1* copy	33	6	18.18%	1.00**	-	-
no rs11547635 & only 1 *TIMP1* copy	128	60	46.88%	3.97	1.63–11.22	0.005
yes rs11547635 & >1 *TIMP1* copy*	2	1	50.00%	-	-	-
yes rs11547635 & only 1 *TIMP1* copy	24	20	80.00%	18.00	5.19–74.89	<0.001
**Groups, outcome of BAV with TAD**	**N**	**N affected**	**% affected**	**OR**	**95% CI**	**P value**
no rs11547635 & >1 *TIMP1* copy	22	2	9.09%	1.00**	-	-
no rs11547635 & only 1 *TIMP1* copy	78	20	25.64%	3.45	0.89–22.80	0.115
yes rs11547635 & >1 *TIMP1* copy*	2	0	0.00%	-	-	-
yes rs11547635 & only 1 *TIMP1* copy	16	9	56.25%	12.86	2.57–99.39	0.004

Logistic regression model for the combinatorial effect of have both rs11547635 and one copy of *TIMP1* on the outcome of having a BAV with TAD.

BAV and TAD are defined as having a BAV and at least one z-score ≥ 1.9 (rounded to the 10th decimal place); those without an AR or AAO measurement were excluded.

*For this group, the sample size was too small, so an accurate odds ratio could not be calculated.

N; total number of subjects.

N affected; number of subjects with BAV or BAV and TAD.

** Reference group

## Discussion

Turner syndrome, like all genetic syndromes, is characterized by a primary inherent defect that sensitizes downstream modifier genes to breach a pathologic threshold. Thus, a single triggering event is capable of unleashing a myriad of phenotypic variations. Consistent with this disease model, we found that in Turner syndrome hemizygosity of *TIMP1* due to lack of a complete second X chromosome is associated with genetic variation of its paralogue, *TIMP3* on chromosome 22, synergistically heightening the risk for BAV and TAD, which is the first sign of aneurysm formation.

Given the detailed understanding of the fundamental role of MMPs in thoracic aortic disease, the results of this study have clear biological relevance. In the euploid population there is a significant reduction in TIMP1 and TIMP3 expression in BAV-associated TAA and a highly significant increase in MMP2 and MMP9, which are both regulated by TIMP1 and TIMP3.[16] This results in a considerable MMP/TIMP imbalance in aneurysms compared to control aortas.

We propose that hemizygosity for *TIMP1* is the X chromosome basis for increased susceptibility for BAV and aortopathy in Turner syndrome. This coupled with a SNP-driven decrease in TIMP3 expression synergistically increases risk for both BAV and BAV with TAD. This is consistent with our hypothesis that a gene or genes on Xp interact with autosomal variants that are benign unless expressed on a genetically sensitized background such as that in Turner syndrome. The inherent decrease in TIMP1 in Turner syndrome subjects missing a complete second copy of the X chromosome sensitizes those individuals to decreased TIMP3 expression. In addition, a global methylation profile for Turner syndrome found that the Turner syndrome X chromosome has a unique methylation pattern when compared to the X chromosome of euploid males.[11] Notably, *TIMP1* tends to be hypermethylated in Turner syndrome,[28] which suggests that the expression level may be decreased even beyond the reduction in copy number.

Importantly, *TIMP1* and *TIMP3* have functional redundancy in the aorta. Both exercise inhibitory control over MMP2 and MMP9, which are the two MMPs associated with degradation of the aortic wall. We propose that decreased TIMP1 expression due to a reduction in copy number sensitizes the aorta to MMP-induced damage, but protection is conferred by the expression of TIMP3. Decreased expression of both negates that protection making the aortic wall vulnerable to degradation which can lead to TAD and aneurysm. In addition, TIMPs 1 and 3 are expressed in the aortic valve, where they play a role in valve remodeling,[23] which is a critical activity in the development of the tricuspid aortic valve. This fundamental link between BAV pathogenesis and downstream TAD provides a previously unrecognized mechanism for the heightened risk for aortopathy in Turner syndrome. In a study of 18 women with TS and aortic dissection, 6 cases were available for biochemical analysis, and that study showed a skewed ratio of collagen I to collagen III (normally 30:70%) with 60% collagen I and only 30% collagen III,[29] which could well be the end result of an altered MMP/TIMP activity.

As with all studies of this nature there are some limitations and caveats. The exome sequencing was done on DNA isolated from peripheral blood, so the molecular karyotypes reflect the chromosome composition in that tissue. It is possible that the karyotype in other tissues such as the developing heart may differ, particularly with respect to mosaicism. In addition, our analyses did not include potential effects of the autosomal rearrangements found in some of the study subjects. These were genetically heterogeneous and often in single individuals, so it is unlikely that they would significantly affect the results of this study. Another limitation is that this study did not assess any potential influence of maternal genetic effects, nor did we assess the parent-of-origin of the retained X chromosome.

There is no clear explanation for the strikingly higher prevalence of aortopathy in euploid men compared to women. And, the larger questions regarding the role of the sex chromosome genes in the differential susceptibility to common diseases has received little attention. Bellott and colleagues proposed that dosage differences between X chromosome genes and homologous ancestral genes retained on the Y chromosome may account for phenotypic differences between men and women.[30] Our data supports another model where expressed genes that escape inactivation on the second X chromosome and that are also absent from the Y chromosome (like *TIMP1*) play a role in the frequently observed sex bias in disease.

In conclusion, we propose that aortopathy in Turner syndrome results from an inherent dysregulation of the TIMP/MMP ratio. This imbalance increases risk for both congenital cardiovascular defects and later onset aortic disease. Beyond Turner syndrome, the lack of a second copy of *TIMP1* in euploid males may also explain the increased risk for BAV/TAD compared to euploid females. The findings of this study represent a significant advance in the understanding of the mechanisms underlying aortopathy in Turner syndrome.

## Materials and methods

### Ethical approval

The Turner syndrome cohort was accessed from the National Registry of Genetically-Triggered Thoracic Aortic Aneurysms and Related Conditions (GenTAC).[31] GenTAC study subject recruitment was approved by the institutional review board for each member of the GenTAC investigative team, and informed consent for participation in associated research studies was obtained for each study subject. The project was approved by the Oregon Health & Science University institutional review board. The Danish cohort was approved by the Central Denmark Region Ethical Scientific Committee (#2012-500-12) and registered at ClinicalTrials.gov (#NCT01678274).

### Study populations

GenTAC spent a decade recruiting study subjects with conditions related to thoracic aortic aneurysms, including collection of biospecimens, rigorous evaluation and documentation of clinical data, and collection of follow-up data for longitudinal studies. The majority of subjects enrolled in GenTAC had aorta imaging studies that provide information on aortic dimensions and evaluation of aortic valve status. All images, such as echocardiograms, CT and MRT studies were collected clinically, but transferred to the GenTAC imaging core (ICORE) for re-evaluation by a single cardiac imaging expert for consistency of measurements and interpretation.[32] The discovery cohort for this study was composed of Turner syndrome study subjects of Northern European (non-Finnish) descent. Inclusion criteria included a diagnosis of Turner syndrome, self-reported race as white, ethnicity as non-Hispanic, evaluation for a diagnosis of BAV, and availability of aortic dimension measurements and body morphometrics. The diagnosis of BAV was based on clinical images and interpretations. An additional 53 study subjects from a prospective study in Denmark were used as an independent replication cohort.[33, 34]

### Phenotyping

For the purposes of this study we defined aortopathy (cases) as those having a BAV with or without TAD. In keeping with clinical norms for Turner syndrome, a thoracic aortic dimension z-score ≥ 1.9 was used as the definition of TAD as an indicator of aneurysm formation. All study subjects were confirmed for a diagnosis of Turner syndrome based on either clinical karyotype or exome sequence-based karyotyping. Subjects were phenotyped for presence of a BAV or a normal aortic valve. Our final Turner syndrome discovery cohort was composed of 88 cases (Turner syndrome with BAV) and 100 controls (Turner syndrome with no BAV). Within this cohort, 113 subjects had aortic root (AR) dimensions and 106 subjects had ascending aorta (AAO) dimensions. For the replication cohort 14 had a BAV and 39 had a normal aortic valve. For all subjects AAO and AR diameters were converted into z-scores using methodology that was specifically developed for children and adults with Turner syndrome to correct for the altered longitudinal growth in Turner syndrome.[35] Briefly, the regression equations and coefficients were used to calculate expected aortic dimensions based on body surface area (BSA, Haycock formula) for each individual in the study with a measurement (Eqs 1&2). The z-scores were calculated by comparing expected aortic dimensions to actual aortic dimensions and incorporating the mean squared error (MSE; Eq 3).[35] Expected aortic dimension data points and lines were generated for each z-score.

*Equations*:

$$\text{Aortic}\;\text{Root}\;\text{equation}:\;{\left(\text{expected}\right)}^{2}={\left(1.035+\left(0.589*\text{BSA}\right)+\left(-0.129*{\text{BSA}}^{2}\right)\right)}^{2}$$ (1)

$$\text{Ascending}\;\text{Aorta}\;\text{equation}:\;{\left(\text{expected}\right)}^{2}={\left(0.942+\left(0.593*\text{BSA}\right)+\left(-0.122*{\text{BSA}}^{2}\right)\right)}^{2}$$ (2)

Z-scoreequation:=(√(actualdimension(cm)−√(expecteddimension(cm))/(√(MSE)) (3)

The BSA (m^2^) vs. AR or AAO (cm) for BAV cases (triangle) and BAV controls (square) were plotted. Overlaid on the same plot are the polynomial trend lines for z = 0, z = 1, z = -1, z = 2, z = -2, z = 3, z = -3 (S2 Fig).

### Whole exome sequencing, quality control and data cleaning

In total, 215 genomic DNA samples isolated from peripheral blood were submitted for exome sequencing and the exome capture kit Roche Nimblegen SeqCap EZ was used to prepare the sequencing libraries. Whole exome sequencing (WES) was performed by the NHLBI Resequencing & Genotyping Service at the University of Washington (D. Nickerson, US Federal Government contract number HHSN268201100037C). In summary, 16 samples failed post-sequencing QC and 199 samples passed post-sequencing QC. The average read depth for the targeted exome was 71X, with 86% of the target regions covered at greater than 20X. Reads were mapped to the hg19 UCSC genome build using the Burrows-Wheeler aligner, version 0.7.10. Variants were called using the GATK best practices pipeline, where in the 199 samples, 195,034 variants were called. BAM files and VCF files were transferred to the Maslen lab for evaluation.

Data cleaning and filtering was performed using PLINK v1.90b3g[36], which 1) removed any variants with less than 99% genotyping rate, where 6,815 variants were removed; 2) removed individuals with more than 5% missing genotypes, where no individuals were removed; 3) excluded markers that fail the Hardy-Weinberg equilibrium test using a threshold of 1.0x10^-6^, where 2,334 variants were removed. A principal components analysis (PCA) was performed using the R package SNPRelate to calculate the eigenvectors (EVs) for each subject.[37] Data were prepared for PCA analysis by taking common SNPs (MAF >5%) and pruning out SNPs in linkage disequilibrium with an r^2^ > 0.2, stepping along five SNPs at a time within 50kb windows. We plotted EV1 vs EV2 to look for population outliers (S3A Fig). Population outliers were removed and the analysis was repeated a total of four times until no more outliers remained (S3B Fig). In total, 11 subjects were detected at EV1 < -0.3 and EV2 > 0.3 and were removed from the dataset. Additionally, we use the first three eigenvectors as covariates in most downstream analysis. The final dataset contained 185,885 variants across 188 subjects, providing a total genotyping rate of 0.998084.

### Gene-based statistical analyses

To enhance the probability of identifying an exome-wide significant signal a gene-based burden test, the optimal sequence kernel association test (SKAT-O), was used to evaluate the data.[38] This analysis clusters variants into genes for a gene by phenotype analysis, which improves signal strength for exome data from smaller cohorts as it reduces the multiple testing burden. This state-of-the-art approach is particularly useful for studies of rare disorders such as Turner syndrome. The 185,885 variants which passed QC from the WES pipeline were assigned to their respective genes using hg19_refGene. Variants were allowed to be in more than one gene since the test compares gene burden in the same gene, not between different genes. All analyses included the first three principal component eigenvalues as covariates to adjust for any underlying population structure. First, SKAT-O was used to test for an association with the dichotomous BAV status. Second, SKAT-O was used to test for an association with BAV and aortic diameter z-scores as a proxy for TAD evaluated as a continuous variable. For each analysis, a quantile-quantile (Q-Q) plot was generated to look for departure of the observed p-values from the expected p-values.

### Variant annotation and validation

Combined Annotation Dependent Depletion (CADD) scores were used as a tool for scoring the deleteriousness of the genetic variants identified in exome sequencing data. PHRED-scaled CADD scores integrate multiple annotations into a single metric that outperforms other commonly used algorithms of this type. A CADD score ≥20 indicates that a variant is among the top 1% most deleterious variants in the human genome. We used the recommended cutoff score of ≥15 as our threshold for considering a variant to be likely deleterious. The allele frequency of each variant was queried in the Exome Aggregation Consortium (ExAC) database of exome data from over 60,000 unrelated individuals, from which we used the European non-Finnish population.[39] All variants with alleles that were overrepresented in cases were validated by Sanger sequencing. For the replication cohort, we performed targeted Sanger sequencing of all *TIMP3* exons and followed the same SKAT-O association test as described above.

### Second sex chromosome status determination

X and Y chromosome information from the WES data was used to assess the presence of any second sex chromosome. X and Y SNP plots were generated for each study subject and compared to control reference plots to define the second sex chromosome status for each individual.[40] Alternate allele frequencies from the exome variant calls were used to create SNP plots. Briefly, the alternate allele frequencies were calculated for all variants on the X chromosome and sorted by position for each subject. In R, scatter plots were generated and evaluated for the presence of a second X chromosome. This was repeated for the Y chromosome and the Integrative Genome Viewer (IGV) was used to confirm the presence of Y chromosome reads.[40] Reference plots of a control female with 46,XX karyotype, a control female with 45,X karyotype, and a control male with 46,XY karyotype were generated (S4A Fig). We then generated X and Y chromosome plots for each subject in this study.

In these plots, the X-axis is sorted by position on the X chromosome and the Y-axis is the alternate (ALT) allele frequency. As expected for a 46,XX karyotype, some SNPs are homozygous for the ALT allele (1.0), homozygous for the reference (REF) allele (0.0), or heterozygous for the ALT/REF allele (0.5). In contrast, a 45,X karyotype only has SNPs that are homozygous for the ALT allele, or homozygous for the REF allele because only one copy is present. The 46,XY karyotype looks similar to the 45,X plot, but has SNPs heterozygous for the ALT/REF allele clustered in the captured pseudoautosomal (PAR) region. The presence of any Y chromosome material was confirmed using IGV. Available clinical karyotypes were compared to molecular karyotypes generated from the SNP data and basic second sex chromosome status groups were created to categorize the study subjects. While the majority of subjects were true monosomy 45,X, examples of other karyotypes included Xp deletions, Xq deletions, Xq isochromosomes, and X chromosome rings for their second X chromosome (S4B Fig); mosaicism for the second X chromosome, either 45,X/46,XX or 45,X/47,XXX, or mosaicism for Y chromosome material (S4C Fig), although we were unable to quantify the Y mosaicism level based on the plots.

While most plots were straight forward in their interpretation, some were more complicated. In those cases, the clinical karyotype was relied upon. To assess the mosaicism observed in a large number of subjects, a model was created to predict the percent 45,X mosaicism based on alternate allele frequencies (S5 Fig). The equations from each model were used, where y is the percent 45,X mosaicism and x is the alternate allele frequency. The average of the upper and lower predicted values was used as the final estimate of 45,X mosaicism. The molecular karyotypes and estimated *TIMP1* copy number based on the percentage of cells with a second X chromosome are shown in Table 7.

**Table 7:** Karyotypes of the TS cohort.

Karyotype	N	*TIMP1* copy number
45,X	109	1.0
45,X/46,XY	15	1.0
46,X,i(Xq)	13	1.0
45,X/46,X,i(Xq)	7	1.0
45,X[50%]/47,XXX[50%]*	4	2.0
45,X [50%]/46,X,ring(X)[50%]	2	1.5
46,X,ring(X), small	2	1.0
45X/46,X,i(Xq)/47,XXX*	1	2.0
46,X,ring(Xp11.1q28)[11%]/46,XX[89%]*	1	1.9
45,X[30%]/46,XX[70%]	1	1.7
45,X[30%]/46,XX[70%]	1	1.7
45,X[32%]/46,XX[68%]	1	1.7
45,X[35%]/46,XX[65%]	1	1.7
45,X[41%]/46,XX[59%]	1	1.6
45,X/46,X,del(Xq21.1)*	1	1.5
45,X[55%]/46,XX[45%]	1	1.5
45,X[65%]/46,XX[35%]	1	1.4
45,X[63%]/46,X,del(Xq11.23)[37%]	1	1.4
45,X[64%]/46,X,del(Xq22q24)[36%]	1	1.4
45,X[72%]/46,XX[28%]	1	1.3
45,X[75%]/46,XX[25%]	1	1.3
45,X[74%]/46,del(Xq13.1)[26%]	1	1.3
45,X[75%]/46,X,ring(X)[25%]	1	1.3
45, X[80%]/46,X,ring(X)[20%]	1	1.2
45,X[82%]/46,XX[18%]	1	1.2
45,X[84%]/46,XX[16%]	1	1.2
45,X[85%]/46,XX[15%]	1	1.2
45,X[85%]/46,XX[16%]	1	1.2
45,X[81%]/46,X,psuidic(Xq21)[19%]*	1	1.2
45,X[82%]/46,del(Xq22)[18%]*	1	1.2
45,X[82%]/46,X,del(Xp22.3p11.4)[18%]	1	1.2
45,X[83%]/46,X,ring(X)[17%]	1	1.2
45,X[85%]/46X,ring(X)[15%]	1	1.2
45,X[88%]/46,XX[12%]	1	1.1
45,X[88%]/46,X,del(Xq13.1)[12%]	1	1.1
45,X,add(15)(p11.2)*	1	1.0
45,X/46,X,+mar*	1	1.0
45,X[20%]/46,X,i(Xq)[80%]*	1	1.0
45,X[82%]/46,X,del(Xp)[18%]	1	1.0
45,X [86%]/ 46,X +mar [13%]*	1	1.0

*Clinical karyotype

N; number of subjects

Genes on Xp were evaluated to identify candidates likely to contribute to aortopathy. We hypothesized that an aortopathy gene would be found on Xp, would escape X inactivation in euploid females,[41–43] would be expressed in the aortic wall, and would not be a pseudogene, or have a Y homologue.

### Statistical analysis

To calculate the magnitude of the association between BAV status and aortic z-score, a linear regression model was fit where BAV was the predictor and aortic z-score was the response variable. This was performed separately for both AR z-score and AAO z-score. The mean differences and 95% confidence intervals were generated to accompany p-values. Boxplots for each AR and AAO z-scores were plotted against BAV status.

To investigate if the *TIMP3* paralog *TIMP1* was associated with BAV, a general logistic regression model was performed where *TIMP1* copy number was the categorical predictor, 1 copy and >1 copy of *TIMP1* were the variables, and BAV status or BAV with TAD was the response variable with no BAV serving as the reference. Odds ratios and 95% confidence intervals were generated to accompany p-values.

To investigate the combination of the *TIMP3* variant rs11547635 and *TIMP1* as risk factors for the presence of a BAV or BAV with TAD, four groups were formed: 1) no *TIMP3* rs11547635 and >1 copy of *TIMP1*, 2) with *TIMP3* rs11547635 and >1 copy of *TIMP1*, 3) no *TIMP3* rs11547635 and only 1 copy of *TIMP1*, and 4) with *TIMP3* rs11547635 and only 1 copy of *TIMP1*. Separate general logistic regression models were created to compare these four groups in order to determine their associations with BAV, or the combination of BAV and TAD. Odds ratios and 95% confidence intervals were generated to accompany p-values.

Other physical attributes of Turner syndrome were studied to determine if any were also associated with the *TIMP3* rs11547635 risk allele. Continuous variables (height, weight, body surface area, systolic blood pressure, and diastolic blood pressure) were analyzed using a Student’s two-sample t-test, where the means of those with or without *TIMP3* rs11547635 were compared. Categorical variables (lymphedema, broad chest, webbed neck, primary ovarian insufficiency, hypertension, coarctation of the aorta, bicuspid aortic valve, and any aortic risk factor) were analyzed using a Chi-squared test with Yate’s correction or Fisher’s exact test as appropriate. The same analysis was done using *TIMP1* copy number as the variable.

## Supporting information

S1 Fig Quantile-Quantile (Q-Q) plots for the SKAT-O analyses. Q-Q plots for SKAT-O analysis of BAV and BAV with AR Z-scores, which shows no significant deviation from the normal distribution.
(TIF)

S2 Fig Calculation of aortic z-scores. Plot of BSA (m^2^) vs. AR or AAO (cm) for BAV cases 10 (triangles) and BAV controls (squares) and polynomial trend 11 lines for expected aorta dimensions for each z-score. A) Plot for aortic root dimensions. B) Plot for ascending aorta dimensions.
(TIF)

S3 Fig Principle component analysis. Principal Component Analysis (PCA) plots of WES samples. A) PCA analysis on all 199 subjects in the study, where eigenvector 1 is plotted against eigenvector 2. B) Final PCA plot after samples were removed due to being population outliers. A total of 11 subjects were removed and a total of 188 subjects remained in the study.
(TIF)

S4 Fig X and Y SNP plots for second sex chromosome status determination. X chromosome SNP plots examples. A) Plots of a known controls representing 45,X, 46,XX, and 46,XY. B) Examples plots of Turner syndrome subjects with ring X, iso Xq, mosaic Xq deletion, and Xp deletion for their second X chromosome. C) Example plots of mosaic 45,X/46,XY, 45,X/47,XXX, and 45,X/46,XX.
(TIF)

S5 Fig Modeling of percent mosaicism from alternate allele counts. A model to predict percent mosaicism from alternate allele counts. The table on the left is the expected alternate allele counts and allele frequencies (frq) for each level of X mosaicism. The plots on the right are the corresponding fitted model using this data, where the line is the fitted trend line with its equation. These equations were used to estimate X mosaicism from observed alternate allele frequencies.
(TIF)

S1 Table Genes on Xp ranked according to the potential to be involved in aortopathy. This list includes all genes on Xp and an analysis of their potential to be involved in the aortopathy phenotype. The decision involved X-inactivation status, whether or not the gene was a pseudogene or a Y-chromosome homolog, and the level of expression in the aorta. The genes are ranked by likelihood for being involved in the pathogenesis of aortopathy based on these criteria.
(PDF)

## References

[1] Prakash S, Guo D, Maslen CL, Silberbach M, Milewicz D, Bondy CA, et al Single-nucleotide polymorphism array genotyping is equivalent to metaphase cytogenetics for diagnosis of Turner syndrome. Genet Med. 2014;16(1):53–9. 10.1038/gim.2013.77 23743550 PMC3883919

[2] Gravholt CH, Juul S, Naeraa RW, Hansen J. Prenatal and postnatal prevalence of Turner’s syndrome: a registry study. BMJ. 1996;312(7022):16–21. 8555850 10.1136/bmj.312.7022.16 PMC2349728

[3] Barr M Jr., Oman-Ganes L. Turner syndrome morphology and morphometrics: Cardiac hypoplasia as a cause of midgestation death. Teratology. 2002;66(2):65–72. 10.1002/tera.10064 12210009

[4] Sybert VP. Cardiovascular malformations and complications in Turner syndrome. Pediatrics. 1998;101(1):E11 9417175 10.1542/peds.101.1.e11

[5] Palmer CG, Reichmann A. Chromosomal and clinical findings in 110 females with Turner syndrome. Hum Genet. 1976;35(1):35–49. 1002163 10.1007/BF00295617

[6] Zinn AR, Page DC, Fisher EM. Turner syndrome: the case of the missing sex chromosome. Trends Genet. 1993;9(3):90–3. 8488568 10.1016/0168-9525(93)90230-f

[7] Bondy C, Bakalov VK, Cheng C, Olivieri L, Rosing DR, Arai AE. Bicuspid aortic valve and aortic coarctation are linked to deletion of the X chromosome short arm in Turner syndrome. J Med Genet. 2013;50(10):662–5. 10.1136/jmedgenet-2013-101720 23825392 PMC3786649

[8] Prakash SK, Bondy CA, Maslen CL, Silberbach M, Lin AE, Perrone L, et al Autosomal and X chromosome structural variants are associated with congenital heart defects in Turner syndrome: The NHLBI GenTAC registry. Am J Med Genet A. 2016;170(12):3157–64. 10.1002/ajmg.a.37953 27604636 PMC5115959

[9] Wang L, Ming Wang L, Chen W, Chen X. Bicuspid Aortic Valve: A Review of its Genetics and Clinical Significance. J Heart Valve Dis. 2016;25(5):568–73. 28238238

[10] Huntington K, Hunter AG, Chan KL. A prospective study to assess the frequency of familial clustering of congenital bicuspid aortic valve. J Am Coll Cardiol. 1997;30(7):1809–12. 9385911 10.1016/s0735-1097(97)00372-0

[11] Silberbach M. Bicuspid aortic valve and thoracic aortic aneurysm: toward a unified theory. J Am Coll Cardiol. 2009;53(24):2296–7. 10.1016/j.jacc.2009.03.028 19520255

[12] Garg V, Muth AN, Ransom JF, Schluterman MK, Barnes R, King IN, et al Mutations in NOTCH1 cause aortic valve disease. Nature. 2005;437(7056):270–4. 10.1038/nature03940 16025100

[13] Shi LM, Tao JW, Qiu XB, Wang J, Yuan F, Xu L, et al GATA5 loss-of-function mutations associated with congenital bicuspid aortic valve. Int J Mol Med. 2014;33(5):1219–26. 10.3892/ijmm.2014.1700 24638895

[14] Qu XK, Qiu XB, Yuan F, Wang J, Zhao CM, Liu XY, et al A novel NKX2.5 loss-of-function mutation associated with congenital bicuspid aortic valve. Am J Cardiol. 2014;114(12):1891–5. 10.1016/j.amjcard.2014.09.028 25438918

[15] Sybert VP, McCauley E. Turner’s syndrome. N Engl J Med. 2004;351(12):1227–38. 10.1056/NEJMra030360 15371580

[16] Rabkin SW. Differential expression of MMP-2, MMP-9 and TIMP proteins in thoracic aortic aneurysm—comparison with and without bicuspid aortic valve: a meta-analysis. Vasa. 2014;43(6):433–42. 10.1024/0301-1526/a000390 25339161

[17] Zhang X, Wu D, Choi JC, Minard CG, Hou X, Coselli JS, et al Matrix metalloproteinase levels in chronic thoracic aortic dissection. J Surg Res. 2014;189(2):348–58. 10.1016/j.jss.2014.03.027 24746253 PMC4065027

[18] Jackson HW, Defamie V, Waterhouse P, Khokha R. TIMPs: versatile extracellular regulators in cancer. Nat Rev Cancer. 2017;17(1):38–53. 10.1038/nrc.2016.115 27932800

[19] Bashash M, Shah A, Hislop G, Treml M, Bretherick K, Janoo-Gilani R, et al Genetic polymorphisms at TIMP3 are associated with survival of adenocarcinoma of the gastroesophageal junction. PLoS One. 2013;8(3):e59157 10.1371/journal.pone.0059157 23527119 PMC3602604

[20] Su CW, Huang YW, Chen MK, Su SC, Yang SF, Lin CW. Polymorphisms and Plasma Levels of Tissue Inhibitor of Metalloproteinase-3: Impact on Genetic Susceptibility and Clinical Outcome of Oral Cancer. Medicine. 2015;94(46):e2092 10.1097/MD.0000000000002092 26579821 PMC4652830

[21] Anderson CL, Brown CJ. Polymorphic X-chromosome inactivation of the human TIMP1 gene. Am J Hum Genet. 1999;65(3):699–708. 10.1086/302556 10441576 PMC1377976

[22] Baker AH, Edwards DR, Murphy G. Metalloproteinase inhibitors: biological actions and therapeutic opportunities. J Cell Sci. 2002;115(Pt 19):3719–27. 12235282 10.1242/jcs.00063

[23] Dreger SA, Taylor PM, Allen SP, Yacoub MH. Profile and localization of matrix metalloproteinases (MMPs) and their tissue inhibitors (TIMPs) in human heart valves. J Heart Valve Dis. 2002;11(6):875–80; discussion 80. 12479292

[24] Ikonomidis JS, Gibson WC, Butler JE, McClister DM, Sweterlitsch SE, Thompson RP, et al Effects of deletion of the tissue inhibitor of matrix metalloproteinases-1 gene on the progression of murine thoracic aortic aneurysms. Circulation. 2004;110(11 Suppl 1):II268–73. 10.1161/01.CIR.0000138384.68947.20 15364874

[25] Silence J, Collen D, Lijnen HR. Reduced atherosclerotic plaque but enhanced aneurysm formation in mice with inactivation of the tissue inhibitor of metalloproteinase-1 (TIMP-1) gene. Circulation Research. 2002;90(8):897–903. 11988491 10.1161/01.res.0000016501.56641.83

[26] Koullias GJ, Ravichandran P, Korkolis DP, Rimm DL, Elefteriades JA. Increased tissue microarray matrix metalloproteinase expression favors proteolysis in thoracic aortic aneurysms and dissections. Annals of Thoracic Surgery. 2004;78(6):2106–10; discussion 10–1. 10.1016/j.athoracsur.2004.05.088 15561045

[27] Allaire E, Forough R, Clowes M, Starcher B, Clowes AW. Local overexpression of TIMP-1 prevents aortic aneurysm degeneration and rupture in a rat model. Journal of Clinical Investigation. 1998;102(7):1413–20. 10.1172/JCI2909 9769334 PMC508989

[28] Trolle C, Nielsen MM, Skakkebaek A, Lamy P, Vang S, Hedegaard J, et al Widespread DNA hypomethylation and differential gene expression in Turner syndrome. Sci Rep. 2016;6:34220 10.1038/srep34220 27687697 PMC5043230

[29] Gravholt CH, Landin-Wilhelmsen K, Stochholm K, Hjerrild BE, Ledet T, Djurhuus CB, et al Clinical and epidemiological description of aortic dissection in Turner’s syndrome. Cardiology in the Young. 2006;16(5):430–6. 10.1017/S1047951106000928 16984695

[30] Bellott DW, Hughes JF, Skaletsky H, Brown LG, Pyntikova T, Cho TJ, et al Mammalian Y chromosomes retain widely expressed dosage-sensitive regulators. Nature. 2014;508(7497):494–9. 10.1038/nature13206 24759411 PMC4139287

[31] Weinsaft JW, Devereux RB, Preiss LR, Feher A, Roman MJ, Basson CT, et al Aortic Dissection in Patients With Genetically Mediated Aneurysms: Incidence and Predictors in the GenTAC Registry. J Am Coll Cardiol. 2016;67(23):2744–54. 10.1016/j.jacc.2016.03.570 27282895 PMC5040186

[32] Asch FM, Yuriditsky E, Prakash SK, Roman MJ, Weinsaft JW, Weissman G, et al The Need for Standardized Methods for Measuring the Aorta: Multimodality Core Lab Experience From the GenTAC Registry. JACC Cardiovasc Imaging. 2016;9(3):219–26. 10.1016/j.jcmg.2015.06.023 26897684 PMC4788536

[33] Mortensen KH, Erlandsen M, Andersen NH, Gravholt CH. Prediction of aortic dilation in Turner syndrome—the use of serial cardiovascular magnetic resonance. J Cardiovasc Magn Reson. 2013;15:47 10.1186/1532-429X-15-47 23742092 PMC3702474

[34] Mortensen KH, Hjerrild BE, Stochholm K, Andersen NH, Sorensen KE, Lundorf E, et al Dilation of the ascending aorta in Turner syndrome—a prospective cardiovascular magnetic resonance study. J Cardiovasc Magn Reson. 2011;13:24 10.1186/1532-429X-13-24 21527014 PMC3118376

[35] Quezada E, Lapidus J, Shaughnessy R, Chen Z, Silberbach M. Aortic dimensions in Turner syndrome. Am J Med Genet A. 2015;167A(11):2527–32. 10.1002/ajmg.a.37208 26118429

[36] Chang CC, Chow CC, Tellier LC, Vattikuti S, Purcell SM, Lee JJ. Second-generation PLINK: rising to the challenge of larger and richer datasets. Gigascience. 2015;4:7 10.1186/s13742-015-0047-8 25722852 PMC4342193

[37] Zheng X, Levine D, Shen J, Gogarten SM, Laurie C, Weir BS. A high-performance computing toolset for relatedness and principal component analysis of SNP data. Bioinformatics. 2012;28(24):3326–8. 10.1093/bioinformatics/bts606 23060615 PMC3519454

[38] Wu MC, Lee S, Cai T, Li Y, Boehnke M, Lin X. Rare-variant association testing for sequencing data with the sequence kernel association test. Am J Hum Genet. 2011;89(1):82–93. 10.1016/j.ajhg.2011.05.029 21737059 PMC3135811

[39] Song W, Gardner SA, Hovhannisyan H, Natalizio A, Weymouth KS, Chen W, et al Exploring the landscape of pathogenic genetic variation in the ExAC population database: insights of relevance to variant classification. Genet Med. 2016;18(8):850–4. 10.1038/gim.2015.180 26681313

[40] Thorvaldsdottir H, Robinson JT, Mesirov JP. Integrative Genomics Viewer (IGV): high-performance genomics data visualization and exploration. Brief Bioinform. 2013;14(2):178–92. 10.1093/bib/bbs017 22517427 PMC3603213

[41] Balaton BP, Cotton AM, Brown CJ. Derivation of consensus inactivation status for X-linked genes from genome-wide studies. Biol Sex Differ. 2015;6:35 10.1186/s13293-015-0053-7 26719789 PMC4696107

[42] Esposito T, Gianfrancesco F, Ciccodicola A, D’Esposito M, Nagaraja R, Mazzarella R, et al Escape from X inactivation of two new genes associated with DXS6974E and DXS7020E. Genomics. 1997;43(2):183–90. 10.1006/geno.1997.4797 9244435

[43] Peeters SB, Cotton AM, Brown CJ. Variable escape from X-chromosome inactivation: identifying factors that tip the scales towards expression. Bioessays. 2014;36(8):746–56. 10.1002/bies.201400032 24913292 PMC4143967

