# Supplementary figures and images for: TIMP3 and TIMP1 are risk genes for bicuspid aortic valve and aortopathy in Turner syndrome
Source: PLoS Genet. 2018 Oct 3;14(10):e1007692. doi: 10.1371/journal.pgen.1007692 (PMC6188895; doi:10.1371/journal.pgen.1007692)

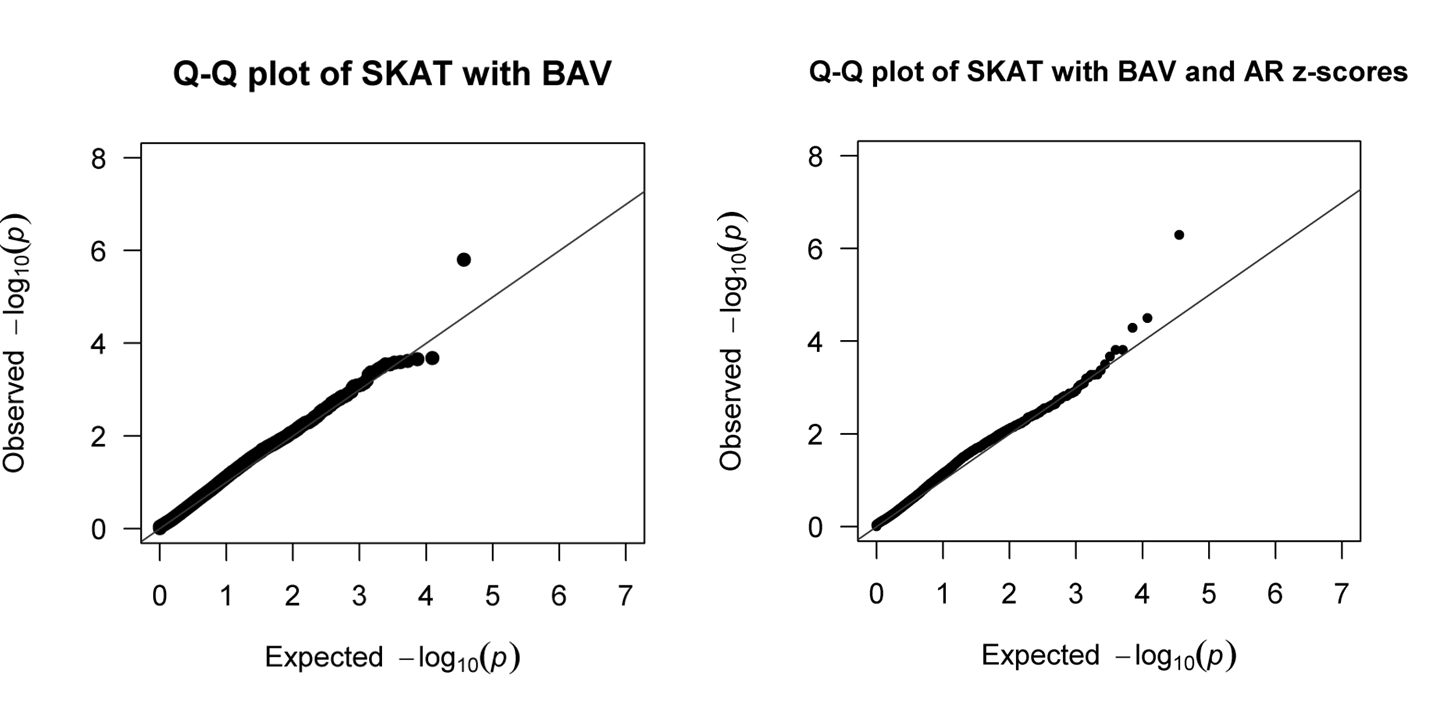

Supplement: S1 Fig — Q-Q plots for SKAT-O analysis of BAV and BAV with AR Z-scores, which shows no significant deviation from the normal distribution. (TIF) [file pgen.1007692.s001.tif]

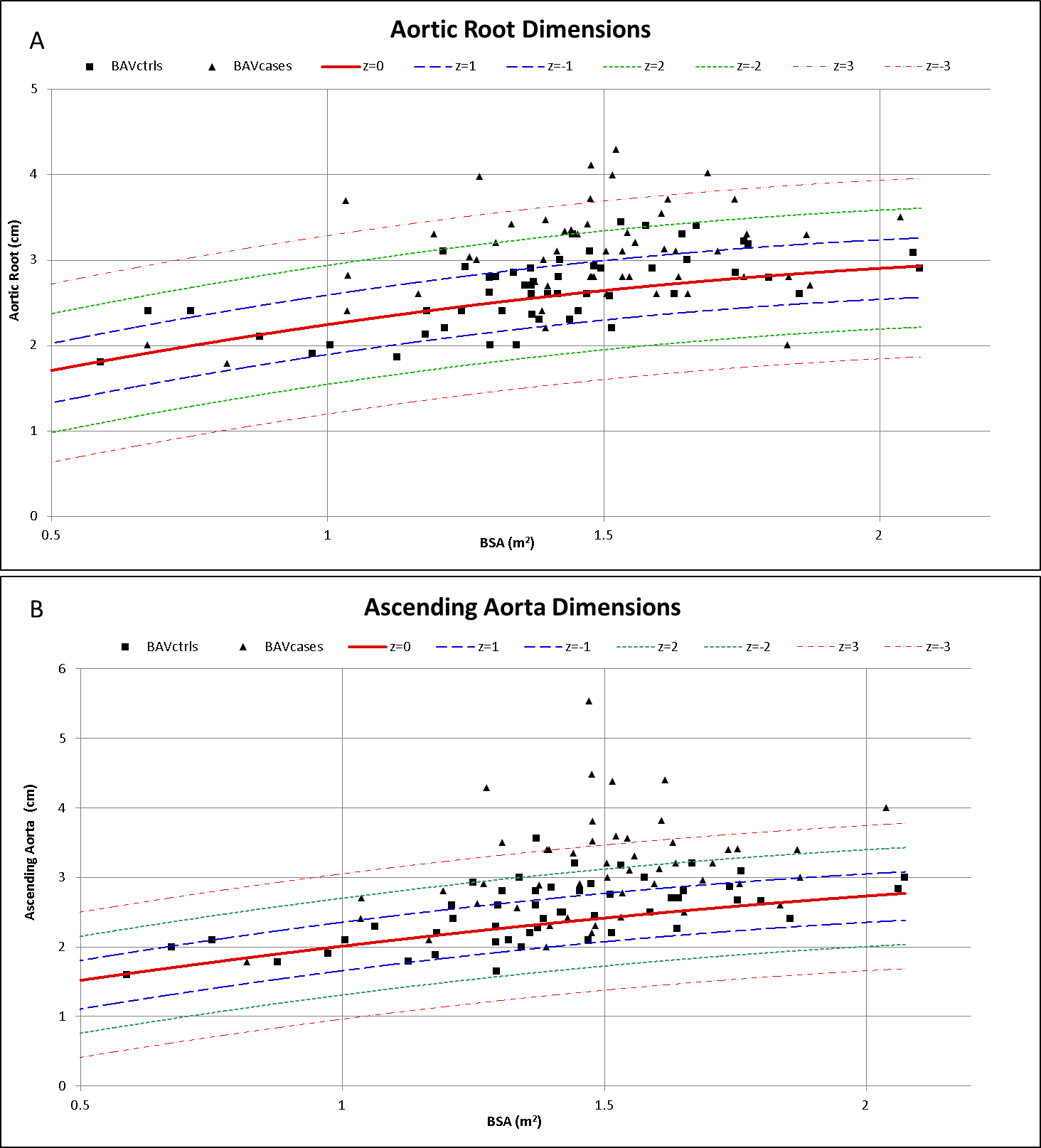

Supplement: S2 Fig — Plot of BSA (m2) vs. AR or AAO (cm) for BAV cases 10 (triangles) and BAV controls (squares) and polynomial trend 11 lines for expected aorta dimensions for each z-score. A) Plot for aortic root dimensions. B) Plot for ascending aorta dimensions. (TIF) [file pgen.1007692.s002.tif]

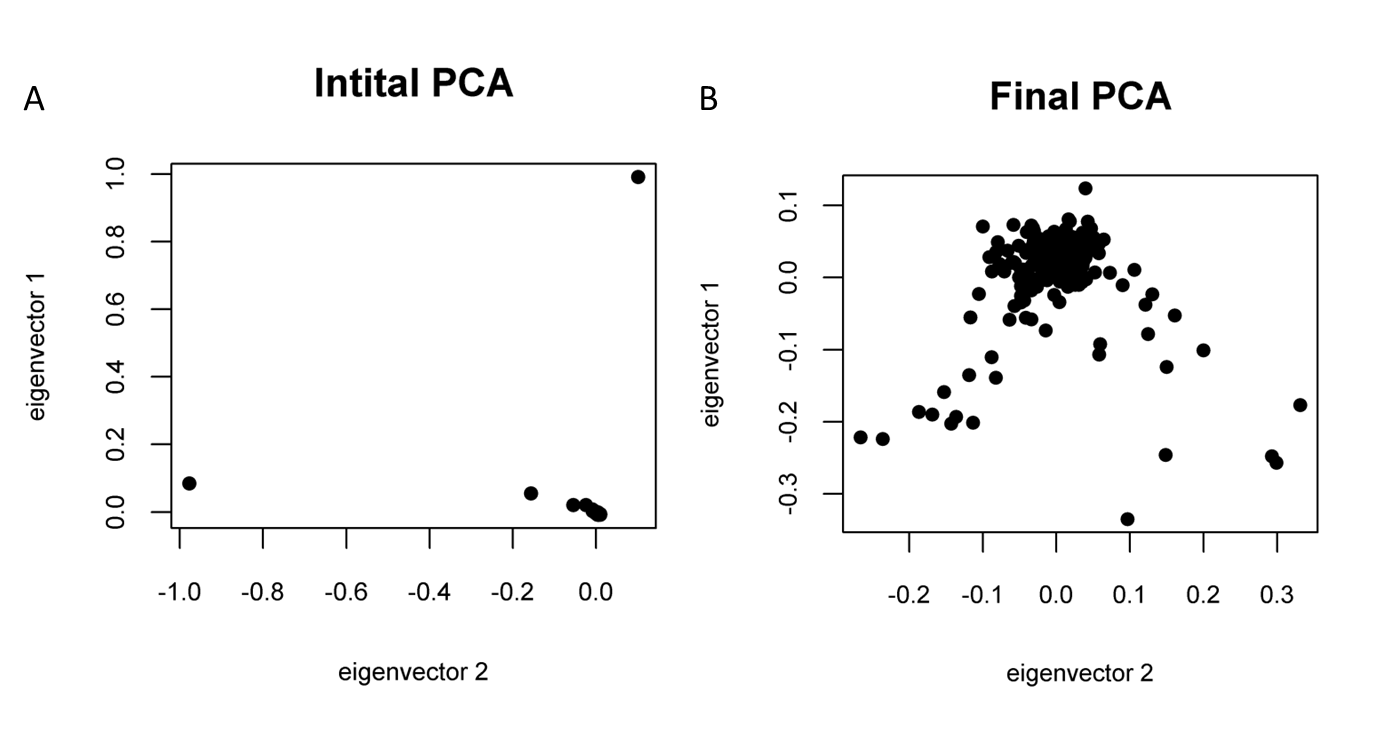

Supplement: S3 Fig — Principal Component Analysis (PCA) plots of WES samples. A) PCA analysis on all 199 subjects in the study, where eigenvector 1 is plotted against eigenvector 2. B) Final PCA plot after samples were removed due to being population outliers. A total of 11 subjects were removed and a total of 188 subjects remained in the study. (TIF) [file pgen.1007692.s003.tif]

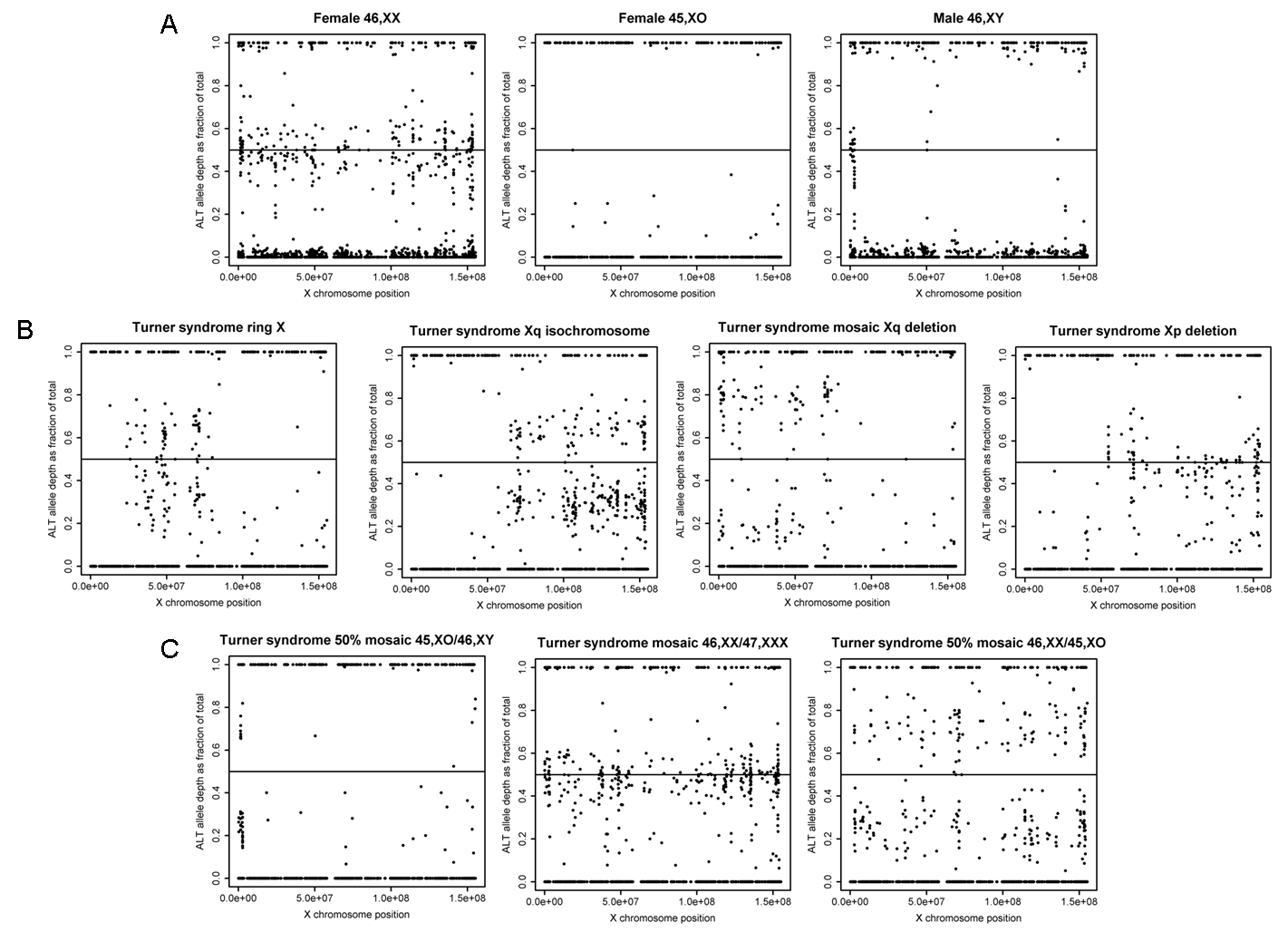

Supplement: S4 Fig — X chromosome SNP plots examples. A) Plots of a known controls representing 45,X, 46,XX, and 46,XY. B) Examples plots of Turner syndrome subjects with ring X, iso Xq, mosaic Xq deletion, and Xp deletion for their second X chromosome. C) Example plots of mosaic 45,X/46,XY, 45,X/47,XXX, and 45,X/46,XX. (TIF) [file pgen.1007692.s004.tif]

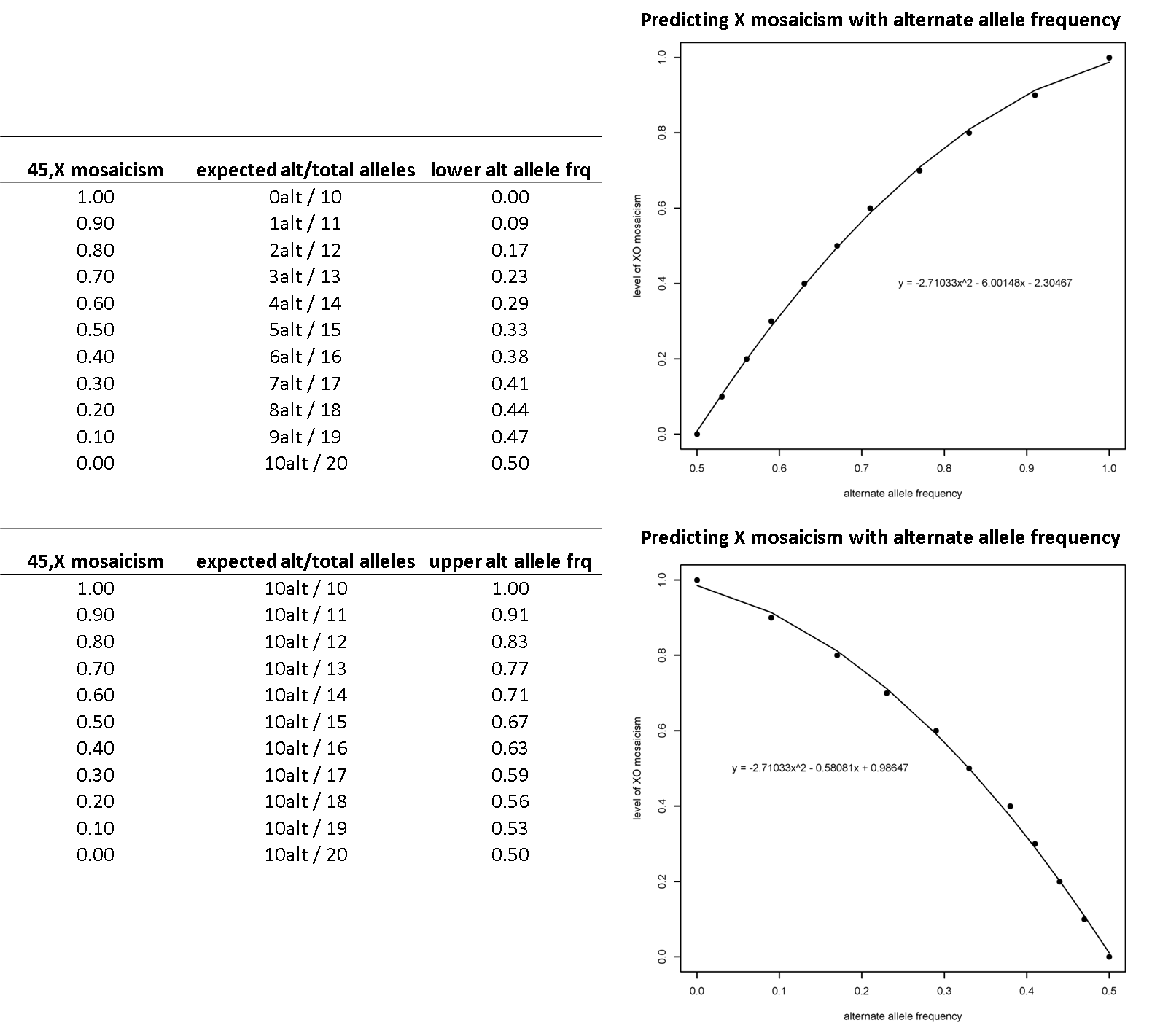

Supplement: S5 Fig — A model to predict percent mosaicism from alternate allele counts. The table on the left is the expected alternate allele counts and allele frequencies (frq) for each level of X mosaicism. The plots on the right are the corresponding fitted model using this data, where the line is the fitted trend line with its equation. These equations were used to estimate X mosaicism from observed alternate allele frequencies. (TIF) [file pgen.1007692.s005.tif]
