# Supplementary material for: TIMP3 and TIMP1 are risk genes for bicuspid aortic valve and aortopathy in Turner syndrome
Source: PLoS Genet. 2018 Oct 3;14(10):e1007692. doi: 10.1371/journal.pgen.1007692 (PMC6188895; doi:10.1371/journal.pgen.1007692)
Supplement: S1 Table — This list includes all genes on Xp and an analysis of their potential to be involved in the aortopathy phenotype. The decision involved X-inactivation status, whether or not the gene was a pseudogene or a Y-chromosome homolog, and the level of expression in the aorta. The genes are ranked by likelihood for being involved in the pathogenesis of aortopathy based on these criteria. (PDF) [file pgen.1007692.s006.pdf]

S1 Table. Genes on Xp ranked according to potential to be involved in aortopathy

gene	ensembl	cdsStart - cdsEnd*	strand	X inactivation status	aorta expression (GTEx median RPKM)	Pseudogene/ Y homolog	meets criteria	rank	reference
TIMP1	ENSG00000102265	X:47442814-47446090	+	VE	423.6	-	yes	1	Balaton et al. 2015
RBM3	ENSG00000102317	X:48433568-48435474	+	VE	48.96	-	yes	2	Wainer-Katsir et al. 2016
UBA1	ENSG00000130985	X:47058201-47074328	+	E	41.44	-	yes	3	Balaton et al. 2015
INE2	ENSG00000281371	X:15805712-15805712	-	E	12.36	-	yes	4	Esposito et al. 1997
AP1S2	ENSG00000182287	X:15845447-15870647	-	Mostly E	8.701	-	yes	5	Balaton et al. 2015
GEMIN8	ENSG00000046647	X:14027031-14039597	-	Mostly E	6.69	-	yes	6	Balaton et al. 2015
CA5B	ENSG00000169239	X:15768146-15800787	+	VE	5.198	-	yes	7	Peeters et al. 2014
NHS	ENSG00000188158	X:17653686-17750584	+	Mostly VE	4.852	-	yes	8	Balaton et al. 2015
TRAPPC2	ENSG00000196459	X:13732525-13738082	-	Mostly E	4.289	-	yes	9	Balaton et al. 2015
CA5BP1	ENSG00000186312	X:15721474-15721474	+	E	3.813	-	yes	10	Balaton et al. 2015
CTPS2	ENSG00000047230	X:16608915-16721025	-	Mostly E	3.593	-	yes	11	Balaton et al. 2015
INE1	ENSG00000224975	X:47065254-47065254	+	E	2.09	-	yes	12	Balaton et al. 2015
TCEANC	ENSG00000176896	X:13680627-13681683	+	Mostly E	0.4852	-	yes	13	Balaton et al. 2015
PPEF1	ENSG00000086717	X:18725899-18845605	+	VE	0.05353	-	yes	14	Balaton et al. 2015
GRPR	ENSG00000126010	X:16142076-16170768	+	Mostly VE	0.02957	-	yes	15	Balaton et al. 2015
FAM9C	ENSG00000187268	X:13056559-13061908	-	E	0.005627	-	yes	16	Balaton et al. 2015
KRBOX4	ENSG00000147121	X:46309895-46332447	+	No call	3.478	-	yes	-	na
BMX	ENSG00000102010	X:15526476-15574270	+	No call	2.041	-	yes	-	Balaton et al. 2015
USP27X	ENSG00000242013	X:49644910-49646227	+	No call	1.952	-	yes	-	Balaton et al. 2015
FAM156B	ENSG00000179304	X:52936252-52936894	+	No call	0.7911	-	yes	-	Balaton et al. 2015
NDP	ENSG00000124479	X:43809044-43817891	-	No call	0.137	-	yes	-	Balaton et al. 2015
ERAS	ENSG00000187682	X:48687533-48688235	+	No call	0.08968	-	yes	-	Balaton et al. 2015
GPR82	ENSG00000171657	X:41586279-41587290	+	No call	0.07618	-	yes	-	Balaton et al. 2015
PAGE2B	ENSG00000238269	X:55102474-55105231	+	No call	0.07481	-	yes	-	Balaton et al. 2015
KLHL34	ENSG00000185915	X:21673971-21675906	-	No call	0.02192	-	yes	-	Balaton et al. 2015
ALAS2	ENSG00000158578	X:55035612-55054240	-	I	0.1066	Y pseudogene	no	-	Balaton et al. 2015
GPKOW	ENSG00000068394	X:48970558-48980072	-	Mostly I	13.43	-	no	-	Balaton et al. 2015
GPM6B	ENSG00000046653	X:13792662-13956629	-	Mostly E	3.678	Y pseudogene	no	-	Balaton et al. 2015
GPR143	ENSG00000101850	X:9693785-9733857	-	Mostly I	0.0387	Y pseudogene	no	-	Balaton et al. 2015
GPR173	ENSG00000184194	X:53105803-53106925	+	Mostly I	2.854	Y pseudogene	no	-	Balaton et al. 2015
GPR34	ENSG00000171659	X:41554886-41556032	+	I	3.703	Y pseudogene	no	-	Balaton et al. 2015
GRIPAP1	ENSG00000068400	X:48830604-48858640	-	I	9.056	Y pseudogene	no	-	Balaton et al. 2015
GSPT2	ENSG00000189369	X:51486722-51488609	+	I	2.974	Y pseudogene	no	-	Balaton et al. 2015
GYG2	ENSG00000056998	X:2748229-2799254	+	Mostly E	0.6903	Y pseudogene	no	-	Balaton et al. 2015
AMELX	ENSG00000125363	X:11312908-11318732	+	No call	0	Y homolog	no	-	Balaton et al. 2015
HCCS	ENSG00000004961	X:11130180-11139930	+	I	5.128	-	no	-	Balaton et al. 2015
HDAC6	ENSG00000094631	X:48660339-48683022	+	Mostly I	14.55	-	no	-	Balaton et al. 2015
HSD17B10	ENSG00000072506	X:53458351-53461292	-	Mostly I	41.21	-	no	-	Balaton et al. 2015
HUWE1	ENSG00000086758	X:53560269-53681051	-	Mostly I	13.56	Y pseudogene	no	-	Balaton et al. 2015
IL1RAPL1	ENSG00000169306	X:28807460-29973937	+	Discordant	0.00543	-	no	-	Balaton et al. 2015
IQSEC2	ENSG00000124313	X:53263400-53350321	-	Mostly E	3.511	Y pseudogene	no	-	Balaton et al. 2015
ITIH6	ENSG00000102313	X:54776327-54824643	-	No call	0	Y pseudogene	no	-	Balaton et al. 2015
KCND1	ENSG00000102057	X:48819841-48826678	-	Mostly I	1.774	Y pseudogene	no	-	Balaton et al. 2015
KDM5C	ENSG00000126012	X:53221925-53254071	-	Mostly E	14.17	Y homolog	no	-	Balaton et al. 2015
KDM6A	ENSG00000147050	X:44732797-44970656	+	Mostly E	3.678	Y homolog	no	-	Balaton et al. 2015
KLF8	ENSG00000102349	X:56259767-56310761	+	I	4.441	-	no	-	Balaton et al. 2015
KLHL15	ENSG00000174010	X:24006037-24024810	-	I	1.847	Y pseudogene	no	-	Balaton et al. 2015
LANCL3	ENSG00000147036	X:37431123-37535066	+	Mostly I	0.2512	Y pseudogene	no	-	Balaton et al. 2015
MAGEB1	ENSG00000214107	X:30268610-30269654	+	No call	0	pseudogene	no	-	Balaton et al. 2015
MAGEB10	ENSG00000177689	X:27839423-27840467	+	No call	0	pseudogene	no	-	Balaton et al. 2015
MAGEB16	ENSG00000189023	X:35820313-35821288	+	No call	0	pseudogene	no	-	Balaton et al. 2015
MAGEB18	ENSG00000176774	X:26157102-26158134	+	No call	0	pseudogene	no	-	Balaton et al. 2015
APEX2	ENSG00000169188	X:55029485-55033868	+	I	5.31	Y pseudogene	no	-	Balaton et al. 2015
MAGEB2	ENSG00000099399	X:30236697-30237657	+	No call	0	pseudogene	no	-	Balaton et al. 2015
MAGEB4	ENSG00000120289	X:30260252-30261293	+	No call	0	pseudogene	no	-	Balaton et al. 2015
MAGED1	ENSG00000179222	X:51637400-51645026	+	I	23.63	pseudogene	no	-	Balaton et al. 2015
MAGED2	ENSG00000102316	X:54835764-54842115	+	Mostly I	99	pseudogene	no	-	Balaton et al. 2015
MAGED4	ENSG00000154545	X:51805325-51811268	-	No call	0	pseudogene	no	-	Balaton et al. 2015
MAGED4B	ENSG00000187243	X:51805124-51811268	-	No call	0	pseudogene	no	-	Balaton et al. 2015
MAGEH1	ENSG00000187601	X:55478807-55479467	+	I	26.74	pseudogene	no	-	Balaton et al. 2015
MAGIX	ENSG00000017621	X:49019227-49022738	+	Mostly I	0.8064	-	no	-	Balaton et al. 2015
MAOA	ENSG00000189221	X:43515589-43603760	+	Mostly I	27.46	-	no	-	Balaton et al. 2015
MAOB	ENSG00000069535	X:43626712-43741545	-	I	40.03	Y pseudogene	no	-	Balaton et al. 2015
APOO	ENSG00000184831	X:23858458-23925819	-	I	2.997	Y pseudogene	no	-	Balaton et al. 2015
MAP3K15	ENSG00000180815	X:19378866-19533379	-	Mostly I	0.02838	Y pseudogene	no	-	Balaton et al. 2015
MAP7D2	ENSG00000184368	X:20028920-20134997	-	Discordant	0.04491	-	no	-	Balaton et al. 2015
MBTPS2	ENSG00000012174	X:21857852-21900773	+	I	2.398	-	no	-	Balaton et al. 2015
MED14	ENSG00000180182	X:40511057-40594664	-	VE	5.1	Y pseudogene	no	-	Balaton et al. 2015
MID1	ENSG00000101871	X:10417407-10535587	-	Mostly I	4.311	Y pseudogene	no	-	Balaton et al. 2015
MID1IP1	ENSG00000165175	X:38664199-38664751	+	I	15.32	-	no	-	Balaton et al. 2015
MOSPD2	ENSG00000130150	X:14910954-14937931	+	I	3.298	-	no	-	Balaton et al. 2015
MSL3	ENSG00000005302	X:11776826-11793198	+	Discordant	5.731	-	no	-	Balaton et al. 2015
MXRA5	ENSG00000101825	X:3227756-3261874	-	Mostly E	1.064	Y pseudogene	no	-	Balaton et al. 2015
ARAF	ENSG00000078061	X:47422366-47430856	+	I	21.52	-	no	-	Balaton et al. 2015
NDUFB11	ENSG00000147123	X:47001715-47004078	-	I	54.46	-	no	-	Balaton et al. 2015
NLGN4X	ENSG00000146938	X:5810857-6069507	-	Mostly E	0.2674	Y homolog	no	-	Balaton et al. 2015
NR0B1	ENSG00000169297	X:30322695-30327480	-	I	0.07369	Y pseudogene	no	-	Balaton et al. 2015
NUDT10	ENSG00000122824	X:51075817-51079091	+	I	1.814	-	no	-	Balaton et al. 2015
NUDT11	ENSG00000196368	X:51234600-51239296	-	I	1.929	-	no	-	Balaton et al. 2015

NYX	ENSG00000188937	X:41307142-41334152	+	I	0	Y pseudogene	no	-	Balaton et al. 2015
OFD1	ENSG00000046651	X:13753190-13787227	+	Mostly E	5.859	Y pseudogene	no	-	Balaton et al. 2015
OTC	ENSG00000036473	X:38211949-38280335	+	I	0.08684	-	no	-	Balaton et al. 2015
OTUD5	ENSG00000068308	X:48780450-48814832	-	I	23.5	-	no	-	Balaton et al. 2015
ARHGAP6	ENSG00000047648	X:11156982-11445715	-	Mostly I	2.977	Y pseudogene	no	-	Balaton et al. 2015
PAGE1	ENSG00000068985	X:49452143-49459373	-	No call	0	-	no	-	Balaton et al. 2015
PAGE2	ENSG00000234068	X:55116453-55119164	+	No call	0	-	no	-	Balaton et al. 2015
PAGE3	ENSG00000204279	X:55284938-55290336	-	No call	0	-	no	-	Balaton et al. 2015
PAGE4	ENSG00000101951	X:49595031-49598465	+	No call	0	-	no	-	Balaton et al. 2015
PAGE5	ENSG00000158639	X:55247815-55250436	+	No call	0	-	no	-	Balaton et al. 2015
PCSK1N	ENSG00000102109	X:48689669-48693934	-	I	0.4771	-	no	-	Balaton et al. 2015
PCYT1B	ENSG00000102230	X:24580409-24690749	-	I	0.1175	-	no	-	Balaton et al. 2015
PDHA1	ENSG00000131828	X:19362155-19377771	+	I	13.55	-	no	-	Balaton et al. 2015
PDK3	ENSG00000067992	X:24483572-24557281	+	I	8.612	Y pseudogene	no	-	Balaton et al. 2015
ARSD	ENSG00000006756	X:2825311-2847316	-	Mostly E	3.108	Y pseudogene	no	-	Balaton et al. 2015
PFKFB1	ENSG00000158571	X:54959835-55020440	-	Discordant	0.05055	-	no	-	Balaton et al. 2015
PHEX	ENSG00000102174	X:22051123-22266070	+	VE	0.09849	Y pseudogene	no	-	Balaton et al. 2015
PHF8	ENSG00000172943	X:53965590-54071323	-	I	5.737	-	no	-	Balaton et al. 2015
PIGA	ENSG00000165195	X:15339627-15350052	-	I	2.354	-	no	-	Balaton et al. 2015
PIM2	ENSG00000102096	X:48771407-48776111	-	I	3.03	-	no	-	Balaton et al. 2015
PIR	ENSG00000087842	X:15403125-15509380	-	Discordant	6.254	-	no	-	Balaton et al. 2015
PLP2	ENSG00000102007	X:49028347-49031043	+	I	156.5	-	no	-	Balaton et al. 2015
PNPLA4	ENSG00000006757	X:7868726-7894160	-	E	1.957	Y pseudogene	no	-	Balaton et al. 2015
POLA1	ENSG00000101868	X:24712078-25014067	+	I	2.033	Y pseudogene	no	-	Balaton et al. 2015
PORCN	ENSG00000102312	X:48369759-48378864	+	Mostly I	5.671	-	no	-	Balaton et al. 2015
ARSE	ENSG00000157399	X:2852872-2878441	-	Mostly VE	0.5414	Y pseudogene	no	-	Balaton et al. 2015
PPP1R2P9	ENSG00000102055	X:42636721-42637330	-	No call	0	-	no	-	Balaton et al. 2015
PPP1R3F	ENSG00000049769	X:49137899-49143552	+	I	2.319	Y pseudogene	no	-	Balaton et al. 2015
PQBP1	ENSG00000102103	X:48755792-48760361	+	Mostly I	21.11	Y pseudogene	no	-	Balaton et al. 2015
PRAF2	ENSG00000243279	X:48929527-48931646	-	I	33.66	-	no	-	Balaton et al. 2015
PRDX4	ENSG00000123131	X:23685687-23704452	+	I	29.11	-	no	-	Balaton et al. 2015
PRICKLE3	ENSG00000012211	X:49032021-49040294	-	I	3.72	-	no	-	Balaton et al. 2015
PRKX	ENSG00000183943	X:3530240-3631294	-	E	1.573	Y pseudogene	no	-	Balaton et al. 2015
PRPS2	ENSG00000101911	X:12809616-12840915	+	I	6.364	-	no	-	Balaton et al. 2015
PRRG1	ENSG00000130962	X:37265502-37312874	+	Mostly I	2.108	-	no	-	Balaton et al. 2015
ARSF	ENSG00000062096	X:2986141-3030597	+	No call	0	Y pseudogene	no	-	Balaton et al. 2015
PTCHD1	ENSG00000165186	X:23352992-23412302	+	Discordant	0.1065	Y pseudogene	no	-	Balaton et al. 2015
RAB9A	ENSG00000123595	X:13726865-13727471	+	Mostly E	9.465	Y pseudogene	no	-	Balaton et al. 2015
RAI2	ENSG00000131831	X:17818537-17820130	-	I	18.99	Y pseudogene	no	-	Balaton et al. 2015
RBBP7	ENSG00000102054	X:16863157-16887764	-	Mostly E	36.21	Y pseudogene	no	-	Balaton et al. 2015
RBM10	ENSG00000182872	X:47006880-47045998	+	I	16.59	-	no	-	Balaton et al. 2015
REPS2	ENSG00000169891	X:16964984-17165604	+	Discordant	1.153	Y pseudogene	no	-	Balaton et al. 2015
RGN	ENSG00000130988	X:46940702-46952346	+	I	8.734	Y pseudogene	no	-	Balaton et al. 2015
RIBC1	ENSG00000158423	X:53453224-53457936	+	Discordant	0.2786	-	no	-	Balaton et al. 2015
RP2	ENSG00000102218	X:46696535-46739204	+	I	3.092	-	no	-	Balaton et al. 2015
ARSH	ENSG00000205667	X:2924653-2951426	+	E	0	Y pseudogene	no	-	Balaton et al. 2015
RPGR	ENSG00000156313	X:38128878-38186620	-	I	1.333	-	no	-	Balaton et al. 2015
RPS6KA3	ENSG00000177189	X:20173515-20284750	-	I	6.236	Y pseudogene	no	-	Balaton et al. 2015
RRAGB	ENSG00000083750	X:55744760-55784776	+	Mostly I	6.93	-	no	-	Balaton et al. 2015
RS1	ENSG00000102104	X:18660123-18690188	-	Discordant	0.02515	-	no	-	Balaton et al. 2015
SAT1	ENSG00000130066	X:23801468-23803973	+	I	111.4	-	no	-	Balaton et al. 2015
SCML1	ENSG00000047634	X:17768073-17771512	+	I	5.658	-	no	-	Balaton et al. 2015
SCML2	ENSG00000102098	X:18259370-18352191	-	Mostly I	0.7291	Y pseudogene	no	-	Balaton et al. 2015
SH3KBP1	ENSG00000147010	X:19554534-19817802	-	Mostly I	3.876	-	no	-	Balaton et al. 2015
SHROOM2	ENSG00000146950	X:9900818-9914977	+	Mostly VE	0.1019	Y pseudogene	no	-	Balaton et al. 2015
SLC35A2	ENSG00000102100	X:48761866-48768913	-	I	5.188	Y pseudogene	no	-	Balaton et al. 2015
ARX	ENSG00000004848	X:25022786-25033854	-	No call	0	-	no	-	Balaton et al. 2015
SLC38A5	ENSG00000017483	X:48317318-48326311	-	I	1.633	-	no	-	Balaton et al. 2015
SLC9A7	ENSG00000065923	X:46466386-46618464	-	I	0.4142	-	no	-	Balaton et al. 2015
SMC1A	ENSG00000072501	X:53407023-53448887	-	Mostly E	7.624	Y pseudogene	no	-	Balaton et al. 2015
SMPX	ENSG00000091482	X:21755680-21772408	-	I	1.259	Y pseudogene	no	-	Balaton et al. 2015
SMS	ENSG00000102172	X:21958942-22012469	+	I	21.7	-	no	-	Balaton et al. 2015
SPACA5	ENSG00000171489	X:47867200-47869042	+	Mostly I	0	-	no	-	Balaton et al. 2015
SPANXN5	ENSG00000204363	X:52825527-52826388	-	No call	0	-	no	-	Balaton et al. 2015
SPIN2A	ENSG00000147059	X:57162253-57163030	-	I	0.09364	-	no	-	Balaton et al. 2015
SPIN2B	ENSG00000186787	X:57146285-57147062	-	Mostly I	4.221	-	no	-	Balaton et al. 2015
SPIN3	ENSG00000204271	X:57020603-57021380	-	Mostly I	2.244	-	no	-	Balaton et al. 2015
ASB11	ENSG00000165192	X:15301626-15332608	-	VE	0.007965	Y pseudogene	no	-	Balaton et al. 2015
SRPX	ENSG00000101955	X:38008963-38080045	-	I	23.72	-	no	-	Balaton et al. 2015
SSX1	ENSG00000126752	X:48116676-48125822	+	No call	0	pseudogene	no	-	Balaton et al. 2015
SSX2	ENSG00000241476	X:52727037-52734799	-	No call	0	pseudogene	no	-	Balaton et al. 2015
SSX3	ENSG00000165584	X:48206938-48214684	-	No call	0	pseudogene	no	-	Balaton et al. 2015
SSX4	ENSG00000204645	X:48243494-48251411	+	No call	0	pseudogene	no	-	Balaton et al. 2015
SSX4B	ENSG00000198946	X:48243494-48251411	+	No call	0	pseudogene	no	-	Balaton et al. 2015
SSX5	ENSG00000165583	X:48047066-48054794	-	No call	0	pseudogene	no	-	Balaton et al. 2015
SSX7	ENSG00000187754	X:52674492-52682522	-	No call	0	pseudogene	no	-	Balaton et al. 2015
SSX8	ENSG00000157965	X:52662998-52662998	+	No call	0	pseudogene	no	-	Balaton et al. 2015
SSX9	ENSG00000204648	X:48165614-48165614	-	No call	0	pseudogene	no	-	Balaton et al. 2015
ASB9	ENSG00000102048	X:15262627-15287996	-	I	0.2425	-	no	-	Balaton et al. 2015
STS	ENSG00000101846	X:7137716-7268302	+	E	4.595	Y pseudogene	no	-	Balaton et al. 2015
SUV39H1	ENSG00000101945	X:48554210-48565961	+	I	1.484	-	no	-	Balaton et al. 2015
SYAP1	ENSG00000169895	X:16737847-16778482	+	E	6.311	Y pseudogene	no	-	Balaton et al. 2015
SYN1	ENSG00000008056	X:47432262-47479127	-	Mostly I	0.7171	Y pseudogene	no	-	Balaton et al. 2015

SYP	ENSG00000102003	X:49047893-49056645	-	I	2.096	-	no	-	Balaton et al. 2015
SYTL5	ENSG00000147041	X:37893142-37985983	+	I	0.008209	Y pseudogene	no	-	Balaton et al. 2015
TAB3	ENSG00000157625	X:30849543-30877705	-	I	2.106	Y pseudogene	no	-	Balaton et al. 2015
TBC1D25	ENSG00000068354	X:48398185-48419363	+	I	5.927	Y pseudogene	no	-	Balaton et al. 2015
TBL1X	ENSG00000101849	X:9621626-9684286	+	Discordant	10.36	Y homolog	no	-	Balaton et al. 2015
ATP6AP2	ENSG00000182220	X:40440317-40465007	+	Mostly I	72.61	Y pseudogene	no	-	Balaton et al. 2015
TFE3	ENSG00000068323	X:48887668-48896850	-	Mostly I	32.52	-	no	-	Balaton et al. 2015
TIMM17B	ENSG00000126768	X:48751011-48755032	-	Mostly I	12.29	-	no	-	Balaton et al. 2015
TLR7	ENSG00000196664	X:12885697-12906777	+	I	0.2005	Y pseudogene	no	-	Balaton et al. 2015
TLR8	ENSG00000101916	X:12928504-12940285	+	No call	0.3294	Y pseudogene	no	-	Balaton et al. 2015
TMEM27	ENSG00000147003	X:15646093-15682898	-	Discordant	0.1118	Y pseudogene	no	-	Balaton et al. 2015
TMEM47	ENSG00000147027	X:34648429-34675146	-	I	77.65	-	no	-	Balaton et al. 2015
TMSB4X	ENSG00000205542	X:12994380-12994930	+	No call	860.3	Y homolog	no	-	Balaton et al. 2015
TRO	ENSG00000067445	X:54948679-54957453	+	Mostly I	6.451	Y pseudogene	no	-	Balaton et al. 2015
ATXN3L	ENSG00000123594	X:13336985-13338053	-	No call	0	-	no	-	Balaton et al. 2015
TSPAN7	ENSG00000156298	X:38420799-38546921	+	Mostly I	10.71	-	no	-	Balaton et al. 2015
TSPYL2	ENSG00000184205	X:53111680-53117121	+	I	74.52	Y homolog	no	-	Balaton et al. 2015
TSR2	ENSG00000158526	X:54466854-54470983	+	I	35.62	-	no	-	Balaton et al. 2015
TXLNG	ENSG00000086712	X:16804610-16859889	+	E	4.382	Y homolog	no	-	Balaton et al. 2015
UBQLN2	ENSG00000188021	X:56590306-56592181	+	I	14.35	Y pseudogene	no	-	Balaton et al. 2015
USP51	ENSG00000247746	X:55513236-55515372	-	I	0.4558	-	no	-	Balaton et al. 2015
USP9X	ENSG00000124486	X:40982881-41091777	+	Mostly E	10.44	Y homolog	no	-	Balaton et al. 2015
UXT	ENSG00000126756	X:47511240-47518326	-	I	43.66	-	no	-	Balaton et al. 2015
BCOR	ENSG00000183337	X:39911361-39937182	-	Mostly I	6.959	Y pseudogene	no	-	Balaton et al. 2015
VCX2	ENSG00000177504	X:8138072-8138684	-	No call	0	-	no	-	Balaton et al. 2015
VCX3A	ENSG00000169059	X:6451785-6452538	-	No call	0	-	no	-	Balaton et al. 2015
VCX3B	ENSG00000205642	X:8433491-8434424	+	No call	0	-	no	-	Balaton et al. 2015
VENTXP1	ENSG00000259849	X:26579169-26579169	+	E	0	-	no	-	Balaton et al. 2015
WAS	ENSG00000015285	X:48542242-48549553	+	VE	2.845	Y pseudogene	no	-	Balaton et al. 2015
WDR13	ENSG00000101940	X:48457339-48463420	+	I	22.88	-	no	-	Balaton et al. 2015
WDR45	ENSG00000196998	X:48932461-48935754	-	I	19.17	-	no	-	Balaton et al. 2015
WNK3	ENSG00000196632	X:54224756-54360106	-	I	0.503	-	no	-	Balaton et al. 2015
WWC3	ENSG00000047644	X:10031578-10109541	+	Mostly I	15.16	Y pseudogene	no	-	Balaton et al. 2015
XAGE2	ENSG00000185751	X:52113157-52118734	+	No call	0	-	no	-	Balaton et al. 2015
BEND2	ENSG00000177324	X:18189094-18238870	-	VE	0	Y pseudogene	no	-	Balaton et al. 2015
XAGE3	ENSG00000171402	X:52891646-52896164	-	No call	0	-	no	-	Balaton et al. 2015
XAGE5	ENSG00000171405	X:52841590-52847237	+	No call	0	-	no	-	Balaton et al. 2015
XK	ENSG00000047597	X:37545214-37587715	+	I	0.4991	Y pseudogene	no	-	Balaton et al. 2015
ZFX	ENSG00000005889	X:24190859-24229493	+	E	3.46	Y homolog	no	-	Balaton et al. 2015
ZNF157	ENSG00000147117	X:47230067-47272993	+	Discordant	0.07125	-	no	-	Balaton et al. 2015
ZNF182	ENSG00000147118	X:47835565-47862008	-	I	2.809	-	no	-	Balaton et al. 2015
ZNF41	ENSG00000147124	X:47306828-47315356	-	I	1.753	Y pseudogene	no	-	Balaton et al. 2015
ZNF630	ENSG00000221994	X:47917856-47926274	-	Mostly I	1.573	Y pseudogene	no	-	Balaton et al. 2015
ZNF645	ENSG00000175809	X:22291108-22292386	+	No call	0	-	no	-	Balaton et al. 2015
ZNF674	ENSG00000251192	X:46359277-46401522	-	I	0.548	-	no	-	Balaton et al. 2015
BMP15	ENSG00000130385	X:50653783-50659607	+	No call	0	-	no	-	Balaton et al. 2015
ZNF81	ENSG00000197779	X:47705666-47776031	+	Mostly I	0.7271	Y pseudogene	no	-	Balaton et al. 2015
ZRSR2	ENSG00000169249	X:15808618-15841365	+	E	11.01	Y pseudogene	no	-	Balaton et al. 2015
ZXDA	ENSG00000198205	X:57934454-57936854	-	I	0.9794	Y pseudogene	no	-	Balaton et al. 2015
ZXDB	ENSG00000198455	X:57618481-57620893	+	I	2.612	-	no	-	Balaton et al. 2015
CACNA1F	ENSG00000102001	X:49061596-49089771	-	I	0.05972	Y pseudogene	no	-	Balaton et al. 2015
CASK	ENSG00000147044	X:41379672-41782241	-	I	3.482	Y pseudogene	no	-	Balaton et al. 2015
CCNB3	ENSG00000147082	X:50028163-50094702	+	Mostly I	0.04199	Y pseudogene	no	-	Balaton et al. 2015
CDK16	ENSG00000102225	X:47082494-47088167	+	Mostly E	13.82	Y pseudogene	no	-	Balaton et al. 2015
CDKL5	ENSG00000008086	X:18525216-18671664	+	Mostly I	1.037	Y pseudogene	no	-	Balaton et al. 2015
CENPVL1	ENSG00000223591	X:51425447-51425447	-	No call	na	pseudogene	no	-	Balaton et al. 2015
CFP	ENSG00000126759	X:47483673-47489243	-	I	0.8031	-	no	-	Balaton et al. 2015
CHST7	ENSG00000147119	X:46433366-46434827	+	I	3.535	Y pseudogene	no	-	Balaton et al. 2015
CLCN4	ENSG00000073464	X:10162988-10201624	+	Mostly I	1.005	-	no	-	Balaton et al. 2015
CLCN5	ENSG00000171365	X:49834580-49856876	+	I	1.082	Y pseudogene	no	-	Balaton et al. 2015
CNKSR2	ENSG00000149970	X:21393015-21670639	+	Mostly I	0.3111	Y pseudogene	no	-	Balaton et al. 2015
CXXC1P1	ENSG00000187893	X:47596027-47596027	+	No call	0	-	no	-	Balaton et al. 2015
CYBB	ENSG00000165168	X:37639330-37670170	+	I	4.286	Y pseudogene	no	-	Balaton et al. 2015
DCAF8L1	ENSG00000226372	X:27997648-27999451	-	No call	0	-	no	-	Balaton et al. 2015
DCAF8L2	ENSG00000189186	X:27765012-27766908	+	No call	0	-	no	-	Balaton et al. 2015
DDX3X	ENSG00000215301	X:41193505-41206972	+	E	42.67	Y homolog	no	-	Balaton et al. 2015
DDX53	ENSG00000184735	X:23018174-23020070	+	No call	0	Y pseudogene	no	-	Balaton et al. 2015
DMD	ENSG00000198947	X:31140035-33357382	-	I	8.586	Y pseudogene	no	-	Balaton et al. 2015
DUSP21	ENSG00000189037	X:44703378-44703951	+	No call	0	-	no	-	Balaton et al. 2015
DYNLT3	ENSG00000165169	X:37699827-37706763	-	I	19.7	-	no	-	Balaton et al. 2015
EBP	ENSG00000147155	X:48382159-48386845	+	Mostly I	4.078	-	no	-	Balaton et al. 2015
EFHC2	ENSG00000183690	X:44008040-44202834	-	Discordant	0.1048	-	no	-	Balaton et al. 2015
EGFL6	ENSG00000198759	X:13587980-13651214	+	I	0.05223	Y pseudogene	no	-	Balaton et al. 2015
EIF1AX	ENSG00000173674	X:20146423-20159758	-	E	14.81	Y homolog	no	-	Balaton et al. 2015
EIF2S3	ENSG00000130741	X:24073085-24094902	+	E	29.13	Y pseudogene	no	-	Balaton et al. 2015
ELK1	ENSG00000126767	X:47496227-47500840	-	I	7.936	-	no	-	Balaton et al. 2015
FAAH2	ENSG00000165591	X:57313258-57515365	+	I	0.1474	-	no	-	Balaton et al. 2015
FAM104B	ENSG00000182518	X:55170211-55187589	-	I	2.956	-	no	-	Balaton et al. 2015
FAM120C	ENSG00000184083	X:54099656-54209631	-	I	1.319	-	no	-	Balaton et al. 2015
FAM156A	ENSG00000182646	X:52977154-52977796	-	I	0.6475	-	no	-	Balaton et al. 2015
FAM47A	ENSG00000185448	X:34148019-34150395	-	No call	0	-	no	-	Balaton et al. 2015
FAM47B	ENSG00000189132	X:34960948-34962886	+	No call	0	-	no	-	Balaton et al. 2015
FAM47C	ENSG00000198173	X:37026483-37029591	+	No call	0	-	no	-	Balaton et al. 2015

FAM9A	ENSG00000183304	X:8759351-8768212	-	No call	0	-	no	-	Balaton et al. 2015
FAM9B	ENSG00000177138	X:8993555-9001027	-	No call	0	-	no	-	Balaton et al. 2015
ACE2	ENSG00000130234	X:15580027-15619034	-	E	0.08828	Y pseudogene	no	-	Balaton et al. 2015
FANCB	ENSG00000181544	X:14861688-14883632	-	I	0.1498	Y pseudogene	no	-	Balaton et al. 2015
FGD1	ENSG00000102302	X:54472541-54521865	-	Mostly I	4.112	-	no	-	Balaton et al. 2015
FOXP3	ENSG00000049768	X:49107794-49114962	-	I	0.07184	Y pseudogene	no	-	Balaton et al. 2015
FOXR2	ENSG00000189299	X:55650144-55651080	+	No call	0	-	no	-	Balaton et al. 2015
FRMPD4	ENSG00000169933	X:12157090-12738652	+	Discordant	0.003936	Y pseudogene	no	-	Balaton et al. 2015
FTHL17	ENSG00000132446	X:31089518-31090070	-	No call	0	-	no	-	Balaton et al. 2015
FTSJ1	ENSG00000068438	X:48337501-48341406	+	I	10.96	Y pseudogene	no	-	Balaton et al. 2015
FUNDC1	ENSG00000069509	X:44383443-44402078	-	E	6.166	Y pseudogene	no	-	Balaton et al. 2015
GAGE1	ENSG00000205777	X:49364777-49370618	+	No call	0	pseudogene	no	-	Balaton et al. 2015
GAGE10	ENSG00000215274	X:49161338-49176229	+	No call	0.05566	pseudogene	no	-	Balaton et al. 2015
ACOT9	ENSG00000123130	X:23722010-23761261	-	I	12.07	-	no	-	Balaton et al. 2015
GAGE12B	ENSG00000236737	X:49326599-49332717	+	No call	0	pseudogene	no	-	Balaton et al. 2015
GAGE12D	ENSG00000227488	X:49297934-49304050	+	No call	0	pseudogene	no	-	Balaton et al. 2015
GAGE12E	ENSG00000216649	X:49336157-49342266	+	No call	0	pseudogene	no	-	Balaton et al. 2015
GAGE12F	ENSG00000236362	X:49297934-49304050	+	No call	0	pseudogene	no	-	Balaton et al. 2015
GAGE12I	ENSG00000241465	X:49217770-49223849	+	No call	0	pseudogene	no	-	Balaton et al. 2015
GAGE12J	ENSG00000224659	X:49179672-49294494	+	No call	0	pseudogene	no	-	Balaton et al. 2015
GAGE13	ENSG00000237597	X:49189215-49294494	+	No call	0	pseudogene	no	-	Balaton et al. 2015
GAGE2A	ENSG00000189064	X:49198718-49313606	+	No call	0	pseudogene	no	-	Balaton et al. 2015
GAGE2C	ENSG00000236249	X:49198718-49214326	+	No call	0	pseudogene	no	-	Balaton et al. 2015
GAGE2D	ENSG00000240257	X:49198718-49223849	+	No call	0	pseudogene	no	-	Balaton et al. 2015
AKAP4	ENSG00000147081	X:49955602-49965540	-	I	0.008884	Y pseudogene	no	-	Balaton et al. 2015
GAGE2E	ENSG00000205775	X:49236830-49294494	+	No call	0	pseudogene	no	-	Balaton et al. 2015
GATA1	ENSG00000102145	X:48649516-48652571	+	I	0.05679	Y pseudogene	no	-	Balaton et al. 2015
GK	ENSG00000198814	X:30671654-30746859	+	I	0.8608	-	no	-	Balaton et al. 2015
GLOD5	ENSG00000171433	X:48620194-48631851	+	No call	0	-	no	-	Balaton et al. 2015
GLRA2	ENSG00000101958	X:14548179-14748607	+	Mostly I	0	Y pseudogene	no	-	Balaton et al. 2015
GNL3L	ENSG00000130119	X:54558999-54587035	+	Mostly I	2.018	Y pseudogene	no	-	Balaton et al. 2015
KANTR	ENSG00000232593	X:53177190-53177190	+	na	na	-	no	-	na
CENPVL2	ENSG00000283093	X:51425447-51425447	-	No call	na	pseudogene	no	-	na
UBE2E4P	ENSG00000233247	X:14263545-14263545	+	No call	na	pseudogene	no	-	na
ADGRG2	ENSG00000173698	X:19008981-19086952	-	No call	na	-	no	-	na
CFAP47	ENSG00000165164	X:35937916-36403121	+	No call	na	-	no	-	na
CLDN34	ENSG00000234469	X:9935397-9936042	+	No call	na	-	no	-	na
HYPM	ENSG00000187516	X:37850092-37850446	+	No call	na	-	no	-	na
JADE3	ENSG00000102221	X:46844295-46918479	+	No call	na	-	no	-	na
MED14OS	ENSG00000234636	X:40594700-40597510	+	No call	na	-	no	-	na
NBDY	ENSG00000204272	X:56755786-56755993	+	No call	na	-	no	-	na
NLRP2B	ENSG00000215174	X:57706555-57706693	-	No call	na	-	no	-	na
VEGFD	ENSG00000165197	X:15364254-15402068	-	No call	na	-	no	-	na
GAGE12C	ENSG00000237671	X:49297934-49304050	+	No call	0	pseudogene	no	-	na
GAGE12G	ENSG00000215269	X:49217770-49223849	+	No call	0	pseudogene	no	-	na
GAGE12H	ENSG00000224902	X:49297934-49304050	+	No call	0	pseudogene	no	-	na
MAGEB17	ENSG00000182798	X:16188505-16189516	+	No call	0	pseudogene	no	-	na
MPC1L	ENSG00000238205	X:40482817-40483228	+	No call	0	pseudogene	no	-	na
SSX2B	ENSG00000157950	X:52727037-52734799	-	No call	0	pseudogene	no	-	na
SSX6	ENSG00000171483	X:47980068-47980068	+	No call	0	pseudogene	no	-	na
VCX	ENSG00000182583	X:7811244-7812057	+	No call	0	Y homolog	no	-	na
FTH1P18	ENSG00000243048	X:37061095-37061761	-	No call	0	-	no	-	na
SPACA5B	ENSG00000171478	X:47867200-47869042	+	No call	0	-	no	-	na
XAGE1B	ENSG00000204382	X:52240503-52243866	+	No call	0	-	no	-	na
XAGE1E	ENSG00000204375	X:52240503-52243866	+	No call	0	-	no	-	na

*hg19 location
X inactivation status: E=escape, VE variable escape, I=inactivated
